# Cardiac Regeneration: New Insights Into the Frontier of Ischemic Heart Failure Therapy

**DOI:** 10.3389/fbioe.2020.637538

**Published:** 2021-01-27

**Authors:** Andrew S. Riching, Kunhua Song

**Affiliations:** ^1^Division of Cardiology, Department of Medicine, University of Colorado Anschutz Medical Campus, Aurora, CO, United States; ^2^Gates Center for Regenerative Medicine and Stem Cell Biology, University of Colorado Anschutz Medical Campus, Aurora, CO, United States; ^3^The Consortium for Fibrosis Research & Translation, University of Colorado Anschutz Medical Campus, Aurora, CO, United States; ^4^Pharmacology Graduate Program, University of Colorado Anschutz Medical Campus, Aurora, CO, United States

**Keywords:** ischemic heart disease, cardiac reprogramming, cardiomyocyte proliferation, cardiac regeneration, revascularization

## Abstract

Ischemic heart disease is the leading cause of morbidity and mortality in the world. While pharmacological and surgical interventions developed in the late twentieth century drastically improved patient outcomes, mortality rates over the last two decades have begun to plateau. Following ischemic injury, pathological remodeling leads to cardiomyocyte loss and fibrosis leading to impaired heart function. Cardiomyocyte turnover rate in the adult heart is limited, and no clinical therapies currently exist to regenerate cardiomyocytes lost following ischemic injury. In this review, we summarize the progress of therapeutic strategies including revascularization and cell-based interventions to regenerate the heart: transiently inducing cardiomyocyte proliferation and direct reprogramming of fibroblasts into cardiomyocytes. Moreover, we highlight recent mechanistic insights governing these strategies to promote heart regeneration and identify current challenges in translating these approaches to human patients.

## Introduction

Cardiovascular diseases (CVD) are the leading cause of death worldwide, accounting for nearly one-third (~18 million) of all global deaths annually (Roth et al., 2017). Approximately half of all CVD-related deaths are caused by ischemic heart disease (IHD), which results in loss of cardiomyocytes (CMs) within the myocardium leading to ventricular dysfunction and heart failure (HF). Numerous pharmacotherapies, implantable devices, and surgical techniques have been pioneered over the last five decades to salvage the myocardium and prevent pathological remodeling, dramatically improving patient outcomes and quality of life following the development of HF (Benjamin et al., 2017; Jones et al., 2019). However, HF mortality rates have begun to plateau (Roth et al., 2017), likely because existing interventions only slow disease progression but are incapable of reversing it. This stems from the fact that adult mammalian hearts exhibit minimal regenerative capacity due to the relative inability of CMs within the adult heart to reenter the cell cycle, and an apparent absence of a stem cell population that can repopulate all cell types within the heart following injury (Hashimoto et al., 2018; Sadek and Olson, 2020).

**Figure 1:**
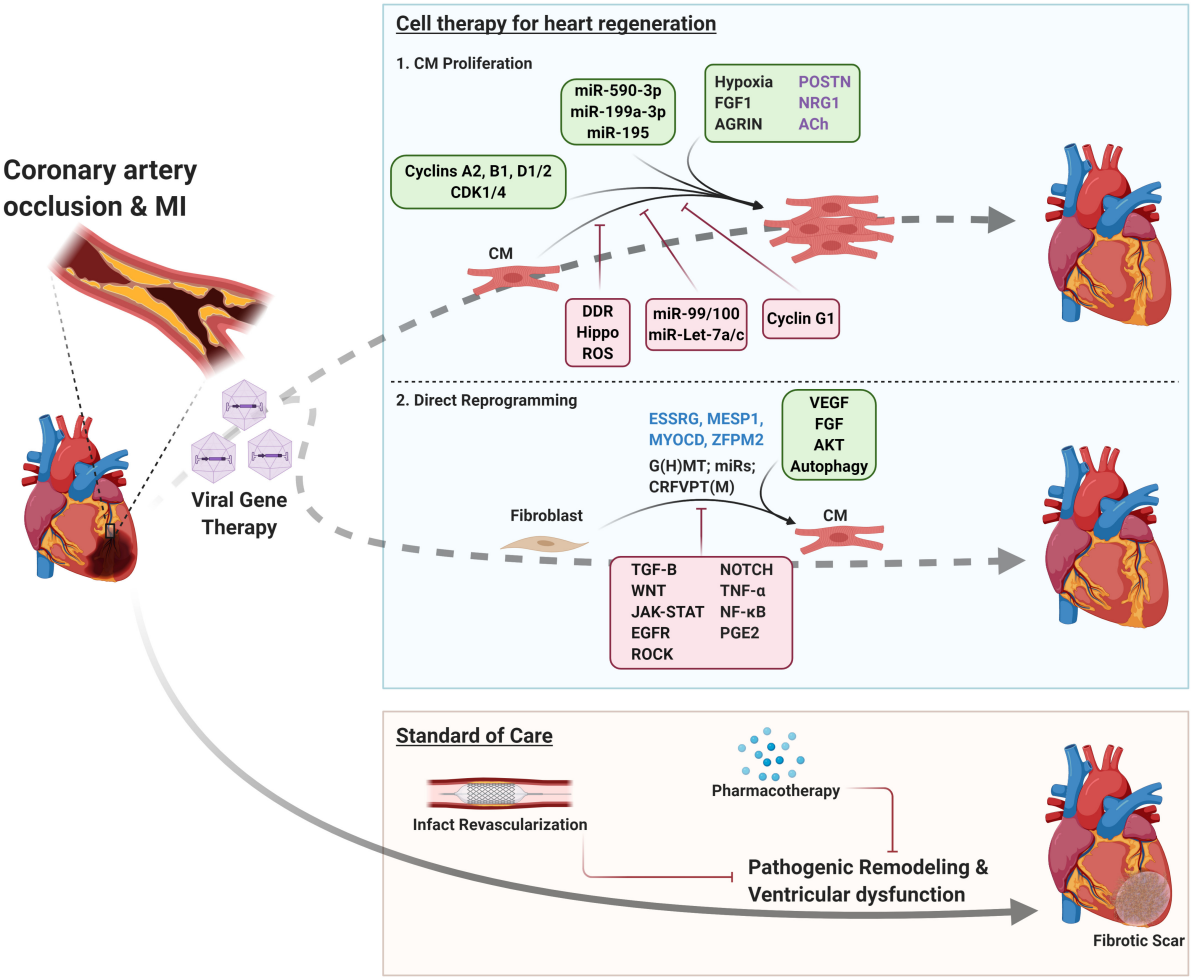
Therapeutic strategies for ischemic heart disease. Tan box; Standard of care: Following MI, patients undergo infarct revascularization and begin pharmacotherapy to slow disease progression, but ultimately develop heart failure and progressive fibrosis. Blue box; Cell therapy for heart regeneration: schematic indicating factors that regulate CM proliferation and direct reprogramming of fibroblasts to CMs to regenerate the myocardium following MI. Green boxes indicate environmental, signaling, and genetic factors that promote CM proliferation/fibroblast reprogramming. Red boxes indicate factors that impair these processes. Factors that have conflicting reports regarding their roles in inducing CM proliferation are listed in purple text. Blue text indicates factors that enhance direct reprogramming in human fibroblasts. DDR, DNA damage response; G(H)MT, *G*ATA4, (*H*AND2), *M*EF2C, *T*BX5; CRFVPT(M), *C*HIR99021, *R*epSox, *F*orskolin, *V*alproic acid, *P*arnate, *T*TNPB, (Rolipra*m*).

Shortly after birth, CMs in mammalian hearts undergo DNA synthesis and cell cycle exit. In rodents, this process occurs within the first few days after birth. Following DNA synthesis, ~75% of rodent CMs undergo nuclear division, resulting in diploid, binucleate CMs. In contrast, the majority of CMs in human hearts remain mononucleate but are tetraploid or polyploid (Laflamme and Murry, 2011). CM ploidy also increases during CM hypertrophy (Laflamme and Murry, 2011), though it remains unclear if increased ploidy is a cause or consequence of hypertrophic growth (Derks and Bergmann, 2020). Until the last two decades, it was generally well-accepted that CMs that exited the cell cycle are quiescent and unable to reenter the cell cycle and complete mitosis. However, more recent evidence based on incorporation of nuclear bomb-derived ^14^C into CM DNA revealed that CM turnover rate in humans is approximately 1% at age 25, and declines to <0.45% past age 75 (Bergmann et al., 2009, 2015). These data indicate that approximately half of the CMs within the heart are generated postnatally (Bergmann et al., 2015). Similarly, it has been observed that CMs in adult mice replenish at a rate of ~0.76%/year, a rate that continues to decline with age (Senyo et al., 2013). Moreover, this rate increases up to 4-fold following myocardial infarction (MI) (Senyo et al., 2013), though whether these new proliferating myocytes arise from existing CMs or cardiac progenitor cells (CPCs) remains somewhat unclear. To determine the origin of these newly proliferating CMs, Hsieh et al. crossed mice harboring a floxed LacZ-polyA sequence followed by GFP under control of the *Actb* promoter with a tamoxifen inducible *Myh6*-Cre mouse line. Adult animals were pulsed with tamoxifen for 14 days to specifically label CMs with GFP while non-myocytes were labeled with LacZ; due to labeling inefficiency, ~82% of CMs expressed GFP while 18% expressed LacZ. During normal aging, these percentages remained relatively constant. However, following MI, the GFP positive percentage of CMs decreased to ~68% whereas the LacZ positive population increased to ~35%, suggesting that a CPC population gave rise to new CMs in response to injury (Hsieh et al., 2007). In contrast, using four independent interleaved reporter lines generating mutually exclusive fluorescent labeling of CMs and non-myocytes by Cre-*lox*P/Dre-*rox* recombinase activity, Li et al. observed that while new myocytes are derived from non-myocytes in embryonic mice, new myocytes in adult mice arise only from existing CMs following MI (Li et al., 2018). Regardless of the origin of new CMs post-MI, the progressive deterioration of heart function and continual fibrotic deposition clearly indicate that the rate at which CMs replenish in adult mammals is insufficient for cardiac regeneration. In this review, we evaluate the progress of three strategies to promote cardiac repair: (1) revascularization, (2) inducing CM proliferation, and 3) direct reprogramming of fibroblasts into CMs (Figure 1). We do not discuss stem cell-based strategies to improve heart function post-MI, as they have been extensively reviewed elsewhere in this special issue (Menasche, 2020).

## Strategies for Cardiac Repair

### Infarct Revascularization

A key development in the treatment of heart failure post-MI was the pioneering of surgical intervention to revascularize the infarcted myocardium via removal of coronary artery occlusions. Evidence that myocardial reperfusion could reduce infarct size dates back to the early 1970s (Ginks et al., 1972), but widespread clinical adaptation of reperfusion was not seen for several more decades. Three primary methods of infarct revascularization remain in use today; enzymatic fibrinolysis/thrombolysis, coronary artery bypass graft (CABG), and percutaneous coronary intervention (PCI; also referred to as percutaneous transluminal coronary angioplasty). Early thrombolysis relied on intravenous delivery of streptokinase (SK) isolated from certain strains of streptococci. The fibrinolytic properties of SK had been described as early as 1933 (Tillett and Garner, 1933), but nearly three decades passed before SK was administered in patients following ST elevation MI (STEMI) (Boucek and Murphy, 1960). Furthermore, it took additional 26 years to establish clinical benefit. The GISSI trial established that administration of SK reduced mortality by 18% compared to control. In a subgroup analysis, SK administration within 3 h of MI exhibited even greater reduction in mortality whereas later administration of SK (9–12 h post-MI) were not associated with reduced mortality [(Gissi), G.I.P.L.S.D.S.N.I.M, 1986]. The TIMI phase I trial demonstrated that recombinant tissue plasminogen activator (rtPA) was nearly twice as effective at recanalizing coronary arteries as SK (Chesebro et al., 1987). Combination therapy of SK and aspirin further reduced mortality compared to SK or aspirin individually [(Isis-2), S.I.S.O.I.S.C.G, 1988]. Thus, these seminal trials demonstrated that myocardial preservation via recanalization could reduce mortality and improve patient outcomes.

Surgical intervention to improve vessel patency also emerged during this period. The first successful coronary vessel graft was performed in 1960 by Robert Goetz in which a patient's right internal thoracic artery was grafted to their right coronary artery. This patient lived an additional 13 months and upon autopsy, the graft was still intact and patent (Goetz et al., 1961). In 1968, the first graft of the left internal thoracic artery to the left anterior descending artery was performed by Green et al. (1968), which set the precedence for the modern CABG procedure (Melly et al., 2018). While CABG remains one of the most performed coronary procedures today with established long-term benefits, it is highly invasive which presents significant risks and discomfort to patients (Melly et al., 2018). Within a decade of George Green's procedure, the first PCI procedures were performed by Andreas Gruntzig by advancing a catheter with an expandable balloon tip through the femoral artery and into an occluded coronary artery in five patients (Gruntzig, 1978). Following PCI, the placement of bare metal stents (BMS) or drug-eluting stents (DES) quickly became the standard of care to combat restenosis (Melly et al., 2018). Stent placement was deemed superior to angioplasty alone for target vessel revascularization and for reducing major adverse cardiac events (Zhu et al., 2001). However, stents themselves can lead to scar formation and restenosis (in the case of BMS) (Weintraub et al., 2013) or thrombus formation (in the case of DES) (Finn et al., 2007). Thus, dual antiplatelet therapy (DAPT), typically consisting of aspirin and P2Y12 receptor blockers (clopidogrel, ticagrelor, and prasugrel), is recommended after stent placement for at least 1 year (reviewed in Weintraub et al., 2013). The AMIHOT II study demonstrated that in conjunction with PCI, intracoronary delivery of supersaturated oxygen (SSO_2_) further reduced infarct size (Stone et al., 2009). A follow-up trial showed that delivery of SSO_2_ via the left main coronary artery for 60 min after PCI in STEMI patients was associated with a favorable safety profile (David et al., 2019). Based on these trials, the FDA approved SSO_2_ therapy in 2019 as an add-on therapy following PCI in STEMI patients.

Many clinical trials have focused on comparing different revascularization strategies to establish which technique should be used in specific patient populations. Based on a meta-analysis of data from 23 trials, PCI and stent placement was deemed superior to thrombolysis at reducing mortality, reinfarction, or stroke in STEMI patients (Keeley et al., 2003). However, not all care facilities are equipped to perform PCI, limiting patients' access. Thus, if patients cannot undergo PCI in a timely manner (within 2 h of acute MI), thrombolysis may be performed instead (O'gara et al., 2013). Following successful revascularization via thrombolysis, transfer to a PCI-capable facility and subsequent catheterization within 24 h of acute MI may further improve revascularization and patient outcome (Al Shammeri and Garcia, 2013; O'gara et al., 2013). Several clinical trials have also focused on comparing PCI to CABG. Though PCI is much less invasive, is associated with lower risks at the time of procedure, and was shown to be non-inferior to CABG in the short term (1–3 years), CABG is associated with superior long-term (>5 years) survival (reviewed in Melly et al., 2018; Ruel et al., 2018). Based on the outcomes from these trials, PCI followed by stent placement and DAPT is the preferred method of revascularization in STEMI patients according to the 2013 ACCF/AHA guidelines. If patients cannot be transferred to a PCI-capable facility in a timely manner, thrombolysis may be performed. Transfer to a PCI-capable facility within 24 h of acute MI may be recommended following successful thrombolysis. CABG is recommended as an alternative to PCI in patients with complex lesions or coronary vasculature intolerant to catheterization (O'gara et al., 2013).

Myocardial reperfusion is estimated to salvage >50% of the myocardium that otherwise would be lost due to infarction (Yellon and Hausenloy, 2007; Frohlich et al., 2013). Furthermore, the advent of revascularization halved in-hospital mortality following acute MI (Braunwald, 2012). However, reperfusion itself causes myocardial damage. It has been postulated that an additional 50 to 80% of the myocardium could be spared if reperfusion injury could be prevented (Yellon and Hausenloy, 2007; Frohlich et al., 2013). Reperfusion injury is likely a significant contributing factor for in-hospital mortality and the development of HF following acute MI (Yellon and Hausenloy, 2007). Oxidative stress, increased intracellular calcium levels, rapid return to physiological pH, and leukocyte invasion have all been linked to causing reperfusion injury in animal models (reviewed in Yellon and Hausenloy, 2007), but targeting these processes has not led to any FDA-approved therapies to date. Thus, exploring new ways to reduce myocardial damage caused by reperfusion may further reduce patient morbidity and mortality.

Infarct revascularization and optimization of pharmacological therapies have radically transformed the treatment and management of post-MI HF. Moreover, these interventions have dramatically reduced mortality, from 5 year mortality rates of 70% in men and 57% in women prior to 1970 (Levy et al., 2002) to <45% between 2000 and 2010 (Benjamin et al., 2017; Jones et al., 2019). It has been argued that numerous pharmacological therapies including renin-angiotensin-aldosterone system inhibitors, dual angiotensin receptor/neprilysin inhibitors, and β-adrenergic receptor antagonists (β-blockers) are significantly underutilized in the HF patient population. Optimizing pharmacotherapy in this population could prevent tens of thousands of deaths per year in the United States alone (Anand, 2018). In contrast, some argue that the strength of β-blocker recommendations in particular should be reevaluated. Most β-blocker trials that demonstrated a reduction in overall mortality were performed prior to reperfusion interventions becoming standard clinical practice following MI. In the reperfusion era, β-blockers have no mortality benefit but are associated with a 30% reduction in recurrent MI and a 20% reduction in angina at the expense of 10% increased risk of worsening heart failure and a 30% increased risk of cardiogenic shock (Bangalore et al., 2014). Risk reduction of recurrent MI and angina appeared to be most significant in the short term (<30 days), thus short-term β-blocker therapy post-MI may maximize benefits while mitigating risks to patients. While the therapies outlined above have proven successful in improving patient survival and slowing HF progression, no current therapy or intervention is able to regenerate CMs lost during MI or reverse cardiac fibrosis.

### CM Proliferation

#### Zebrafish Heart Regeneration

In contrast to mammals, amphibian and teleost CMs are able to reenter the cell cycle, resulting in CM mitosis and partial or full heart regeneration upon injury (reviewed in Kikuchi and Poss, 2012; Porrello and Olson, 2014; Gonzalez-Rosa et al., 2017). Heart regeneration in zebrafish is particularly well-established. Following ~20% resection of the ventricle, a fibrin clot forms to seal the ventricular wound, which is ultimately replaced by proliferating CMs over the course of 60 days (Poss et al., 2002). Heart regeneration has also been observed following cryoinjury, wherein a copper filament is cooled with liquid nitrogen and placed against approximately 25% of the ventricle to induce myocardial damage. In this model, CMs die via necrosis and apoptosis rather being removed surgically, which represents a more physiologically relevant model of myocardial damage. Following cryoinjury, necrotic tissue was replaced by fibrotic scar tissue before being replaced by newly proliferated CMs, resulting in full heart regeneration after ~130 days (Gonzalez-Rosa et al., 2011). A third model of myocardial damage, in which diptheria toxin A chain (DTA) is expressed specifically in CMs via tamoxifen inducible *Myl7*-Cre resulted in ablation of ~60% of ventricular CMs. Remarkably, CM proliferation was induced within 1 week of genetic ablation, resulting in full replenishment of lost CMs and restored heart function (Wang et al., 2011).

Though immunofluorescent staining indicated CMs undergo DNA synthesis and mitosis in these models, whether newly generated CMs arose from existing CMs or differentiated from CPCs remained unclear. Using a tamoxifen inducible GFP reporter under the control of the Cmlc2a (*Myl7*) promoter, Jopling et al. permanently labeled CMs 48 h post-fertilization. Following ventricular resection, regenerated tissue was uniformly GFP positive, indicating new CMs were derived from existing CMs (Jopling et al., 2010). More GFP/ bromodeoxyuridine (BrdU) double positive CMs were detected in injured animals than uninjured controls, which was accompanied by upregulation of the cell cycle progression regulator polo like kinase 1 (Plk1). Moreover, proliferating CMs displayed disassembled sarcomeres and reduced cell-cell contact but did not upregulate Hand2/Nkx2.5 expression indicating only limited CM de-differentiation occurred in response to injury (Jopling et al., 2010). Following ventricular resection, CMs upregulated expression of a *gata4:EGFP* reporter in the subepicardial ventricular layer prior to the injury site. Moreover, EGFP expression preceded induction of DNA synthesis and proliferation. Proliferating CMs initially uncoupled from the conduction system but reintegrated after apical regeneration (Kikuchi et al., 2010). Mechanistically, cell cycle reentry is influenced by cellular signaling (reviewed in Gonzalez-Rosa et al., 2017). For example, following injury, expression of the retinoic acid synthesizing enzyme Raldh2 was induced in the endocardium. Degradation of retinoic acid (RA) by inducible expression of Cyp26a1 or inducing expression of dominant negative Raldh2 reduced CM proliferation by >85%. However, administration of exogenous RA was insufficient to induce proliferation in CMs (Kikuchi et al., 2011). Notch1b and DeltaC were upregulated 1 day following ventricular resection, and remained elevated through day 7 post-amputation, indicating activation of Notch precedes heart regeneration (Raya et al., 2003). Moreover, inhibition of Notch signaling blunted CM proliferation following ventricular resection (Zhao et al., 2014). Finally, Neuregulin 1 (Nrg1) expression was strongly induced following genetic ablation of CMs (Gemberling et al., 2015). Myocardial overexpression of Nrg1 strongly induced CM proliferation, even in the absence of injury, resulting in cardiomegaly and dramatic thickening of the ventricular wall. In contrast, inhibition of Erbb2, an Nrg1 co-receptor, blocked CM proliferation in response to injury (Gemberling et al., 2015). Further elucidation of the mechanisms that induce CM proliferation in zebrafish may lead to novel strategies to induce CM proliferation in humans post-MI.

#### Neonatal Heart Regeneration

Despite limited regeneration in adults, remarkably, a brief window exists during which partial or complete regeneration occurs after injury in neonatal mammals. Neonatal mice exhibit full heart regeneration following resection of 15% of the ventricle 1 day after birth (postnatal day 1; P1) (Porrello et al., 2011b). Troponin T positive CMs in resected hearts displayed significant increases in phosphorylated Histone H3 and Aurora B kinase, markers indicative of mitosis and cytokinesis, respectively. CM proliferation peaked 1 week after resection, and mitotic cells were detected not only at the site of injury, but throughout the heart. To determine the origin of proliferating CMs, Porrello et al. utilized a Rosa26-LacZ reporter under the control of tamoxifen inducible αMHC-Cre to generate LacZ positive CMs. Neonatal animals were dosed a single pulse of tamoxifen at birth to label CMs and apical resection was performed at P1. Regeneration following resection did not change the percentage of LacZ positive cells indicating new CMs were derived from existing CMs, not CPCs. In contrast to resection at P1, resection at P7 instead resulted in the development of a fibrotic scar (Porrello et al., 2011b), coinciding with previous observations that CM binucleation begins at P4 and is followed by cell cycle exit (Li et al., 1996). Additional evidence shows that a second proliferative burst of CMs occurs at P15 in mice, which corresponds to pre-adolescence in humans. During this period, both heart weight and CM number increase by ~40% (Naqvi et al., 2014). Notably, this proliferative burst could be entirely suppressed by inhibiting thyroid hormone (T3) biosynthesis whereas T3 administration doubled the number of BrdU positive cells in isolated CMs, indicating increased DNA synthesis. P15 animals also exhibited partial heart regeneration post-MI; infarction size in P15 animals was smaller than in P21 animals and functional parameters were only modestly reduced in P15 animals but severely compromised in P21 animals (Naqvi et al., 2014). More recently, it has also been demonstrated that P2 pigs exhibit significant cardiac regeneration but P3 or P14 pigs instead form fibrotic scars following MI (Ye et al., 2018). As observed in murine neonates, CMs in P2 pigs undergo DNA synthesis and cytokinesis, evidenced by BrdU incorporation and Aurora B kinase staining. These studies indicate that like amphibians and teleosts, mammalian hearts also possess significant regenerative capacity, but this capacity is lost shortly after birth.

#### Mechanisms Governing Mammalian CM Proliferation

Numerous extracellular factors have been shown to regulate CM proliferation during development and disease. In mice, increased reactive oxygen species (ROS) production and a switch from glycolysis to oxidative phosphorylation occurs shortly after birth, correlating to the interval during which CMs exit the cell cycle. Hypoxia, ROS scavenging, or inhibition of the DNA damage response all prolonged the interval during which postnatal mice hearts could regenerate following injury (Puente et al., 2014). Moreover, fate mapping of hypoxic CMs by fusing the oxygen-dependent degradation domain of HIF-1α to tamoxifen inducible Cre, resulting in fluorescent labeling of hypoxic CMs, indicated that these CMs are more proliferative and tend to be mononucleated compared to normoxic CMs, evidenced by increased labeling by cell cycle and DNA synthesis markers Ki67 and BrdU (Kimura et al., 2015). By RNA-seq, hypoxic CMs upregulated Cyclin-dependent kinase (CDK)/cyclin genes and downregulated negative cell cycle regulators including *Meis1*. Gradual exposure to severe hypoxia (7% O_2_) reduced oxidative phosphorylation and DNA damage post-MI, which coincided with induction of CM proliferation compared to normoxia. Further, hypoxia reduced fibrotic area and improved ventricular function 1 week after infarction (Nakada et al., 2017). Additionally, extracellular matrix (ECM) components such as the fibroblast-derived protein periostin (POSTN) may regulate CM proliferation. Administration of truncated recombinant human POSTN increased BrdU incorporation in adult rat CMs 14-fold compared to non-stimulated CMs (Kuhn et al., 2007). Myocardial delivery of POSTN via Gelfoam patch increased ejection fraction (EF) and reduced scar size 12 weeks post-MI in rats whereas untreated rats showed no functional improvement and displayed extensive scarring. Additionally, the number of CMs undergoing mitosis increased 40-fold in POSTN treated animals. POSTN knockout (KO) impaired myocardial regeneration in neonatal mice, evidenced by increased fibrosis, reduced ventricular function, and reduced CM proliferation 3 weeks post-MI (Chen et al., 2017). In contrast, Lorts et al. did not observe increased DNA synthesis, mitosis, or cytokinesis in neonatal mouse or rat CMs following 2–3 day treatment with full length POSTN. Moreover, neither genetic deletion nor overexpression altered CM proliferation *in vivo* post-MI (Lorts et al., 2009). Thus, the contexts in which POSTN regulates cardiac regeneration and CM proliferation require further exploration. Finally, KO of the ECM protein agrin impaired cardiac regeneration in P1 mice following ventricular resection whereas intramyocardial injection of agrin induced CM cell cycle reentry in juvenile and adult hearts resulting in reduced scar area and significant improvement in ventricular function post-MI (Bassat et al., 2017).

As in zebrafish, diverse signaling pathways and growth factors have been shown to induce CM proliferation in both neonates and adult animals. Inhibition of p38 alongside fibroblast growth factor 1 (FGF1) stimulation improved ventricular function and reduced fibrosis in rats post-MI, which was accompanied by increased CM mitosis and angiogenesis (Engel et al., 2006). Inhibition of p38 alone also increased proliferation of both neonatal and adult rat CMs, and potentiated proliferation induced by other ligands including NRG1, FGF1, and interleukin 1 β (IL-1β) (Engel et al., 2005). Furthermore, CM-specific genetic deletion of p38α nearly doubled the percentage of mitotic CMs in neonatal mice. NRG1 induced proliferation in neonatal rat CMs and improved cell viability and survival in both neonatal and adult rat CMs (Zhao et al., 1998). Additionally, NRG1β induced CM proliferation in mononucleated but not binucleated adult rat and mouse CMs *in vitro* and *in vivo* (Bersell et al., 2009). Genetic lineage tracing confirmed that proliferating CMs arose from existing CMs rather than progenitor cells. Further, NRG1 injection attenuated fibrosis, improved EF, and decreased compensatory hypertrophy post-MI (Bersell et al., 2009). Single or dual administration of NRG1 and FGF1 via microparticle injection improved heart function and reduced fibrosis post-MI in rats (Formiga et al., 2014). Either growth factor alone or in combination also increased vasculogenesis in infarcted hearts, but only NRG1 increased the number of Ki67 positive CMs. A follow up study demonstrated that microparticle delivery of NRG1 and FGF1 also improved ventricle function and infarct vasculogenesis post-MI in pigs (Garbayo et al., 2016). While FGF1 administration did not affect fibrosis, NRG1 administration reduced scar area. In contrast to these studies, Reuter et al. did not observe increased CM DNA synthesis post-infarction in mice administered NRG1, and even observed reduced CM DNA synthesis in the uninjured heart (Reuter et al., 2014). Nevertheless, short term NRG1 administration showed promising trends in improving left ventricular EF (LVEF) and reducing end systolic and diastolic volume in human patients in a phase II clinical trial (Gao et al., 2010). However, whether these favorable trends resulted from increased CM proliferation, vasculogenesis, or additional affects by NRG1 to prevent cardiac remodeling requires further investigation.

Cardiac regeneration requires sympathetic reinnervation following injury. Chemical sympathectomy via 6-Hydroxydopamine hydrobromide (6-OHDA) resulted in extensive fibrosis and a lack of cardiac regeneration in P2 neonatal mice (White et al., 2015). Similarly, transgenic overexpression of Semaphorin3aa resulted in hypo-innervation in zebrafish hearts and greatly impaired regeneration following ventricular resection (Mahmoud et al., 2015). Pharmacological inhibition of cholinergic nerve function by the non-selective muscarinic receptor antagonist atropine or the type 2 muscarinic (M2) receptor-specific antagonist methoctramine impaired heart regeneration and reduced CM proliferation in both zebrafish and neonatal mice. Moreover, removal of the left vagus nerve impaired cardiac regeneration in P1 mice post-MI, which could be rescued by administration of NRG1 and nerve growth factor (NGF) (Mahmoud et al., 2015). Intriguingly, vagal nerve stimulation was found to promote long term survival in rats post-MI (Li et al., 2004). However, disappointingly, the INOVATE-HF trial concluded that vagal nerve stimulation was not associated with increased survival or reduced HF events in humans (Gold et al., 2016).

Suppression of the conserved Hippo signaling pathway has also been demonstrated to induce CM proliferation and regulate heart development. Activation of this pathway triggers a kinase cascade leading to phosphorylation of Yes-associated Protein (YAP), resulting in nuclear exclusion and subsequent degradation. Inactivation of this kinase cascade allows active (non-phosphorylated) YAP to translocate to the nucleus and regulate transcription with its coactivator TEA Domain Transcription Factor 1 (TEAD1). CM-specific KO of *Lats1/2*, which directly phosphorylate YAP, induced CM DNA synthesis, cell cycle reentry, and cytokinesis in adult mouse hearts (Heallen et al., 2013). Similarly, CM-specific KO of *Salvadore* (*Salv*), a scaffold protein that mediates the phosphorylation and activation of LATS1/2 by upstream kinases MST1/2, also induced CM proliferation in adult mice. Both *Salv* and *Lats1/2* KO animals displayed reduced scar area and improved ventricular function following ventricular resection at P8, a time point typically past the regenerative window in neonatal mice. *Salv* KO also reduced fibrotic area and improved ventricular function following coronary artery ligation in adult mice (Heallen et al., 2013; Leach et al., 2017), and resulted in upregulation of an inducer of mitophagy, Parkin (Park2). *Park2* KO blocked regeneration in adult *Salv*-KO mice post-MI (Leach et al., 2017). Paired-like homeodomain transcription factor 2 (Pitx2) was also induced in the border zone of adult mice harboring CM-specific *Salv*-KO post-MI. Pitx2 was observed to co-regulate ROS-related genes along with YAP. Interestingly, double KO of *Pitx2* and *Salv* in adult mice prevented cardiac regeneration post-MI, but regeneration could be restored by treatment with the antioxidant N-acetyl cysteine (Tao et al., 2016). *Salv* KO driven by *Nkx2-5*-Cre resulted in cardiomegaly in neonatal mice and increased phosphorylation of Histone H3 during development (Heallen et al., 2011). In support of these studies, *Yap1* KO (Von Gise et al., 2012) or dual KO of *Yap1* and *Taz* (Xin et al., 2013) resulted in embryonic lethality due to severe ventricular hypoplasia. *Yap1* KO alongside *Taz* haploinsufficiency resulted in viable embryos, but surviving animals exhibited markedly reduced CM proliferation and developed severe lethal cardiomyopathy within the first 2 weeks of life (Xin et al., 2013). Transgenic overexpression of constitutively active (phospho-null) YAP S112A induced CM proliferation post-MI in P7 and P49 mice resulting in reduced fibrosis and improved ventricular function (Xin et al., 2013). Similarly, overexpression of S127A YAP induced CM proliferation in embryonic mice and in isolated neonatal CMs, coinciding with induction of cell cycle genes *Ccna2, Ccnb1*, and *Cdc2* (Von Gise et al., 2012). Mice overexpressing constitutively active YAP developed lethal cardiomegaly due to CM hyperplasia. These pro-proliferative effects were mediated via the interaction between YAP and TEAD1. Embryonic mice expressing YAP S79A, a mutation that abolished the interaction between YAP and TEAD1, exhibited marked hypoplasia, comparable to *Yap1* KO embryos (Von Gise et al., 2012).

Forced expression of cyclins and CDK proteins have shown promise in inducing CM cell cycle reentry. Embryonic mice specifically overexpressing Cyclin A2 in CMs led to cardiomyocyte hyperplasia and cardiomegaly (Chaudhry et al., 2004). CMs retained proliferative potential after birth, demonstrated by significantly elevated proliferating cell nuclear antigen (PCNA) and phosphorylated Histone H3 signal compared to control animals at P7 and P14, which corresponded to a greater population of mononucleated CMs (Chaudhry et al., 2004). Moreover, Cyclin A2 adenoviral overexpression improved LVEF in adult pigs post-MI, which was accompanied by increased percentages of CMs positive for phosphorylated Histone H3 and Ki-67 (Shapiro et al., 2014). Further, Cyclin A2 overexpression reduced the collagen/muscle density ratio, indicating reduced fibrosis vs. Adeno-Null control. Transgenic overexpression of Cyclin D2, but not Cyclin D1 or D3 resulted in DNA synthesis following cardiac injury in mice, which correlated to reduced infarct size and increased CM numbers detected within the infarct 5 months post-MI (Pasumarthi et al., 2005). The newly regenerated myocardium electrically coupled with the surrounding heart tissue, and cardiac function in Cyclin D2 transgenic animals was indistinguishable from sham-operated mice 6 months post-MI whereas heart function in control animals continued to decline (Hassink et al., 2008). Overexpression of the combination of CDK1, CDK4, Cyclin B1, and Cyclin D1 resulted in cell division in up to 20% of adult CMs expressing all four proteins (Mohamed et al., 2018). Moreover, adenoviral intramyocardial delivery of these cell cycle regulators before or 1 week after coronary artery ligation reduced scar size and resulted in near complete restoration of heart function 12 weeks post-MI whereas animals injected with control virus displayed extensive fibrotic scarring and poor heart function. Additionally, CDK1 and Cyclin B1 overexpression could be replaced by pharmacologic inhibition of Wee1 and transforming growth factor (TGF)-β (Mohamed et al., 2018). Silencing of cell cycle proteins in adult CMs is facilitated by Retinoblastoma (Rb) and Retinoblastoma-like protein 2 (p130). CM-specific KO of *Rb1* in p130 deficient mice impaired recruitment of heterochromatin protein 1 (HP1)-γ, resulting in de-repression of cell cycle genes including *Cyce1, E2f1, Cycb1, cdc2, cdc25c, Plk1*, and *Aurkb*, which coincided with increased cytokinesis in isolated adult CMs (Sdek et al., 2011). In contrast, overexpression of Cyclin G1 induced DNA synthesis and nuclear division, but inhibited cytokinesis in neonatal rat CMs, thereby inducing polyploidy but not proliferation (Liu et al., 2010). Acute pressure overload by transverse aortic constriction (TAC) induces CM hypertrophy and increases polynucleation of CMs. However, *Ccng1* KO mice did not exhibit increased numbers of polynucleated CMs after TAC (Liu et al., 2010). Thus, overexpression of Cyclins A2, B1, and D2, and CDK1 and 4 promote CM cell cycle reentry while expression of Cyclin G1 instead seems to play a role in binucleation.

Recently, several groups have established the involvement of microRNAs (miRs) in regulating cell cycle and proliferation in CMs. Eulalio et al. utilized high content microscopy to identify 204 miRs that induced proliferation in neonatal rat CMs, evidenced by triple staining with Ki67, EdU, and α-actinin (Eulalio et al., 2012). Two of these candidate miRs (miR-590-3p and miR-199a-3p) also induced proliferation in adult rat CMs. Transcriptome profiling by RNA-seq revealed these miRs upregulated expression of genes related to cell cycle, cell growth and proliferation, and DNA replication, recombination, and repair. Additionally, *in vivo* transfection or adeno-associated virus (AAV)9-mediated intramyocardial delivery of these miRs increased ventricular wall thickness and resulted in gross enlargement of neonatal rat hearts, coinciding with marked increases in EdU incorporation and H3 phosphorylation in CMs. AAV9 miR injection also preserved ventricular function and reduced scar size following coronary artery ligation, which was accompanied by increased numbers of EdU positive CMs (Eulalio et al., 2012). A follow up study demonstrated that AAV6-miR-199a-1 intramyocardial delivery also improved ventricular function and reduced infarct size and fibrosis after 1 month in a porcine ischemia/reperfusion injury model (Gabisonia et al., 2019). Functional improvement correlated to increased detection of BrdU, Ki67, and phosphorylated H3 in CMs. In contrast, several miRs have also been demonstrated to suppress CM proliferation. CM-specific transgenic overexpression of the miR-15 family member miR-195 resulted in congenital heart defects including ventricular septal defects and right ventricle hypoplasia in neonatal mice (Porrello et al., 2011a). Gross heart size was reduced, and animals that did not die during development developed lethal cardiomyopathy by 5 to 6 months of age. Hearts at P1 had fewer mitotic CMs but increased numbers of binucleated/multinucleated CMs. The authors established that miR-195 directly targets *Chek1*, resulting in failure of CMs to progress through the G2 checkpoint and undergo mitosis. Administration of locked nucleic acid (LNA)-modified anti-miRs targeting both miR-15b and miR-16 resulted in downregulation of all miR-15 family members and extended the proliferative window of CMs in postnatal mice through P12 (Porrello et al., 2011a). CM-specific transgenic overexpression of miR-195 also blunted cardiac regeneration in P1 neonatal mice following coronary artery ligation (Porrello et al., 2013). In contrast, antagonizing miR-195 by administering LNA-modified anti-miRs prior to MI resulted in partial restoration of cardiac function following ischemia/reperfusion injury in P21 mice. Aguirre et al. observed downregulation of ~60 miRs following ventricular resection in zebrafish, including the miR-99/100 and let-7a/c families, which are well-conserved across species (Aguirre et al., 2014). MiR-99/100 mimic resulted in defective heart regeneration whereas antagomiR-99/100 induced strong proliferation and enlargement of the heart in uninjured zebrafish. Notably, these miRs are not downregulated upon injury in mammals. Lentiviral silencing of miR-99/100 and/or miR-Let-7a/c in adult mice induced CM dedifferentiation, evidenced by sarcomere disassembly and upregulation of GATA4, which was accompanied by H3 phosphorylation. These effects were all enhanced by hypoxia. Antagonizing miR-99/100 and miR-Let-7a/c resulted in improved ventricular function, reduced scar size, and increased CM proliferation in adult mice following coronary artery ligation (Aguirre et al., 2014).

While specific mechanisms and contexts during which these diverse factors regulate CM proliferation in response to injury remain elusive, collectively, these studies illustrate that inducing CM proliferation represents a promising strategy to regenerate the myocardium following MI.

### Fibroblast Reprogramming

While CMs occupy an estimated 70% of the ventricular volume (Tang et al., 2009) they account for only 25–35% of the total number of cells residing in the heart (Nag, 1980; Bergmann et al., 2015). The non-myocyte fraction is comprised primarily of endothelial cells, immune cells, and cardiac fibroblasts (CFs), the latter of which comprise ~15% of the non-myocyte cell population and ~11% of the total cells residing in the heart (Pinto et al., 2016). Upon ischemic injury, CFs are activated by biochemical and mechanical cues triggering migration and expansion into the infarct and border zone. Activated CFs secrete extracellular matrix (ECM) proteins to produce a fibrotic scar, which is integral to prevent myocardial rupture following loss of CMs due to ischemia (reviewed in Davis and Molkentin, 2014; Travers et al., 2016; Tallquist and Molkentin, 2017; Humeres and Frangogiannis, 2019). However, persistent CF activation leads to progressive fibrosis, which in turn worsens heart function post-MI. Thus, CFs serve as an ideal pool of cells to reprogram to repopulate the infarct with CMs and mitigate the chronic deposition of ECM.

The first evidence of fibroblast reprogramming was observed in 1987 wherein transfection of a single cDNA containing the coding region of the *MyoD1* gene was found to convert multiple fibroblast cell lines into skeletal muscle myoblasts (Davis et al., 1987). MyoD was later found to induce skeletal muscle differentiation not only in dermal fibroblasts, but also chondroblasts, smooth muscle, and retinal pigmented epithelial cells with varying degrees of efficiency. While retinal pigmented epithelial cells demonstrated lower differentiation efficiency than cells from mesodermal lineages, they still displayed prominent striation, multinucleation, and growth arrest, all key features of skeletal muscle morphogenesis (Choi et al., 1990). Thus, MyoD was established as a “master regulator” of skeletal muscle differentiation.

Unfortunately, to date, no parallel “master regulator” of cardiac muscle differentiation has been identified. However, the landmark discovery that iPSCs can be generated from fibroblasts via transcription factor (TF) overexpression (Takahashi and Yamanaka, 2006; Takahashi et al., 2007) generated interest in identifying combinations of TFs that cooperatively reprogram fibroblasts into terminally differentiated cells including neurons (Vierbuchen et al., 2010), hepatocytes (Huang et al., 2011), and CMs (Ieda et al., 2010). Ieda et al. identified TFs enriched in CMs compared to CFs by microarray and selected 14 which resulted in embryonic lethality due to cardiac defects when mutated for further analysis. Serial removal of individual TFs identified a cardiomyogenic core consisting of GATA4, MEF2C, and TBX5 (GMT) which induced ~4.8% of neonatal CFs to express the cardiac isoform of Troponin T (cTnT). Moreover, a small population of these cTnT^+^ cells exhibited spontaneous calcium transients and visible beating after 4 to 5 weeks in culture (Ieda et al., 2010). GMT transduction generated similar results upon delivery to dermal (tail tip) fibroblasts (TTFs), though the frequency of calcium oscillations was reduced. Subsequent studies confirmed that GMT, though inefficient, is sufficient to reprogram a subpopulation of mouse embryonic fibroblasts (MEFs), neonatal mouse CFs and TTFs, and adult mouse or rat CFs and TTFs both *in vitro* and *in vivo* (Chen et al., 2012; Mathison et al., 2012; Qian et al., 2012; Ma et al., 2015). Table 1 summarizes GMT-based reprogramming strategies.

**Table 1:** *In vitro* and *in vivo* efficiencies of GMT-based reprogramming strategies.

**Reprogramming strategy**			**Fibroblast origin**			**Efficiency**			**Study**
**Core factors**	**Species**	**Additional factors**	**Embryonic**	**Dermal**	**Cardiac**	**Cardiac marker expression**	**Spontaneously beating cells**	**Reprogramming *in vivo***	**Authors**
GMT	Mouse	None		X	X	4% cTnT/αMHC-GFP^+^ (CFs); 2.5% cTnT/αMHC-GFP^+^ (TTFs)	Rare	Rare detection of reprogrammed cells in hearts	Ieda et al., 2010
		Polycistronic delivery			X	3–7% αMHC-GFP^+^	Not discussed	~1% GFP/α-actinin^+^ following GMT+GFP injection into infarct	Inagawa et al., 2012
		+Thymosin β4			X	N/A	N/A	GMT vs. DsRed	Qian et al., 2012
								EF = 25% vs. 16%; Fibrosis reduced 75%	
		None		X	X	35% GFP^+^ Tnnt2-Cre/Rosa26 mTmG reporter TTFs	Not detected	Cardiac gene expression detected in reprogrammed cells	Chen et al., 2012
		+MyoCD, SRF, MESP1, and SMARCD3	X			2.4% αMHC-GFP^+^	Not detected	N/A	Christoforou et al., 2013
		+miR-133	X		X	20% α-actinin^+^, 12% cTnT^+^ (MEFs); 3.5% cTnT^+^ (TTFs)	~7-fold enhanced by miR-133 vs. GMT alone	N/A	Muraoka et al., 2014
		Polycistronic delivery		X	X	3–4% cTnT/αMHC-GFP^+^ (neonatal CFs)	~10-fold enhanced by polycistronic delivery vs. previous studies	N/A	Wang et al., 2015a
		+FGF2/FGF10/VEGF (FFV)	X	X		1% cTnT/αMHC-GFP^+^	~20-fold enhanced by FFV vs. GMT alone	N/A	Yamakawa et al., 2015
		Polycistronic delivery			X	N/A	N/A	Polycistronic GMT vs. DsRed	Ma et al., 2015
								EF = ~38% vs. 17%; FS = ~18.4% vs. ~7.5%; Fibrotic area = ~20% vs. ~40%	
		Polycistronic delivery +shBmi1	X	X	X	22% cTnT/αMHC-GFP^+^ (neonatal CFs)	2-fold enhanced by shBmi1	N/A	Zhou et al., 2016
		Polycistronic delivery +TGF-β/WNT inhibition			X	16% cTnT/αMHC-GFP^+^ (neonatal CFs)	~40% of cells seeded	GMT ± inhibitors vs. DsRed	Mohamed et al., 2017
								EF = ~35% vs. 20%; Fibrotic area = ~15% vs. ~39%	
		Polycistronic delivery +shPbtb1			X	45% cTnT/αMHC-GFP^+^	Not discussed	N/A	Liu et al., 2017
		Polycistronic Sendai viral delivery	X	X	X	10% cTnT^+^	~50% of MEFs seeded; ~10% of total cells	Polycistronic GMT vs. GFP	Miyamoto et al., 2018
								EF = ~35% vs. ~25%; FS = ~15% vs. ~10%; Reduced fibrosis	
		Polycistronic delivery +shBcor	X		X	~9–16% cTnT/αMHC-GFP^+^ (CFs); ~1.2% cTnT/αMHC-GFP^+^ (MEFs)	Not discussed	N/A	Zhou et al., 2018
		Alternate MEF2C isoforms	X	X	X	5% cTnT/αMHC-GFP^+^ (MEFs); 4% cTnT/αMHC-GFP^+^ (CFs); 1.25% cTnT/αMHC-GFP^+^ (TTFs)	Not discussed	N/A	Wang et al., 2020b
		Polycistronic delivery +shBeclin1	X	X	X	6% cTnT^+^ (MEFs); 20% cTnT^+^ (CFs); 5% cTnT+ (TTFs)	~3-fold enhanced by shBeclin1	Polycistronic GMT ± Beclin^±/−^ vs. DsRed	Wang et al., 2020c
								EF = ~40% vs. ~20%; FS = ~29% vs. ~10%; Fibrotic area = 25% vs. 45%	
	Rat	+VEGF		X	X	7% cTnT^+^	Not discussed	GMT ± VEGF vs. GFP	Mathison et al., 2012
								EF = 63% vs. 48%; Fibrotic area = 12% vs. 24%	
		Polycistronic delivery +VEGF			X	7.5% cTnT^+^	Not discussed	GMT ± VEGF vs. GFP;	Mathison et al., 2014
								EF = 48% vs. 39%; Fibrosis = 21% vs. 31%	
		None			X	5% cTnT^+^	Not discussed	N/A	Singh et al., 2016
		+VEGF and adenoviral GMT delivery			X	6.9% cTnT^+^	Not discussed	Ad-GMT±VEGF vs. Ad-Null;	Mathison et al., 2017
								EF (Change from baseline); +21% vs. −0.4%	
		+HDAC/WNT inhibition and Retinoic Acid			X	23% cTnT^+^	Not detected	N/A	Singh et al., 2020

#### Optimizing the Combination of Reprogramming Factors

Although GMT expression is sufficient to induce expression of cardiac sarcomere proteins, the generation of functional induced CMs (iCMs) is inefficient. Thus, numerous studies focused on optimizing the combination of TFs to increase the yield of functional iCMs. The addition of HAND2 to GMT (GHMT) increased the percentage of cells expressing both cTnT and the αMHC-GFP reporter 3- to 5-fold compared to GMT (9.2% vs. 2.9% in adult TTFs, 6.8% vs. 1.4% in adult CFs) (Song et al., 2012). Additionally, ~1% of total cells (~5% of reprogrammed cells) formed well-organized sarcomeres following GHMT transduction (Nam et al., 2014). Intriguingly, the original 14 factor screen performed by Ieda et al. indicated removal of HAND2 enhances reprogramming efficiency (Ieda et al., 2010), thus the role of HAND2 in promoting cardiac reprogramming was initially controversial. However, follow up studies from several groups have conclusively demonstrated that the inclusion of HAND2 markedly improves reprogramming efficiency, at least in part through more efficient upregulation of cardiac TF, ion channel, and sarcomere genes and suppression of cell cycle genes. These genetic changes correlate to more organized sarcomere formation, increased calcium flux, and generation of increased numbers of beating cells (Umei et al., 2017; Zhang et al., 2019a,b; Wang et al., 2020b). GHMT-based reprogramming strategies are summarized in Table 2.

**Table 2:** *In vitro* and *in vivo* efficiencies of GHMT-based reprogramming strategies.

**Reprogramming strategy**			**Fibroblast origin**			**Efficiency**			**Study**
**Core factors**	**Species**	**Additional factors**	**Embryonic**	**Dermal**	**Cardiac**	**Cardiac marker expression**	**Spontaneously beating cells**	**Reprogramming *in vivo***	**Authors**
GHMT	Mouse	None		X	X	6.8% cTnT/αMHC-GFP^+^ (CFs), 9.2% cTnT/αMHC-GFP^+^ (TTFs)	0.2% of iCMs with sarcomeres that also express cTnT, αMHC-GFP	GHMT vs. GFP	Song et al., 2012
								EF = 57% vs. 29% at 12 weeks; Fibrotic area reduced 50%	
		+NKX2.5	X		X	cTnT-GCaMP activity induced in 1.6% of MEFs and 4% of CFs	Rare	N/A	Addis et al., 2013
		Fusion of MyoD transactivation domain to MEF2C	X	X		20.9% cTnT^+^ (MEFs, Day 7), 29.3% cTnT^+^ (TTFs, Day 20)	3.2–3.5% of seeded MEFs; 0.025% of seeded TTFs	N/A	Hirai et al., 2013
		+NKX2.5 and TGF-β inhibition	X		X	cTnT-GCaMP activity induced in 17% of MEFs and 9.3% of CFs	~5-fold enhanced by TGF-β inhibition	N/A	Ifkovits et al., 2014
		None	X	X	X	1.2% sarcomere positive	0.16% of reprogrammed MEFs	N/A	Nam et al., 2014
		Fusion of MyoD transactivation domain to MEF2C + Ezh2 or G9a/GLP inhibition	X			17% cTnT/Hcn4^+^	23% (Ezh2 inhibition) or 20% (G9a/GLP inhibition) of reprogrammed MEFs	N/A	Hirai and Kikyo, 2014
		+miR-1, miR-133, and TGF-β inhibition	X	X	X	64% α-actinin^+^, 67% cTnT^+^ (MEFs); 18% α-actinin^+^, 18% cTnT^+^ (CFs); 30% α-actinin^+^, 35% cTnT^+^ (TTFs)	60% of total cells at Day 11 (MEFs); 2.5% of total cells (CFs); 4% of total (TTFs)	N/A	Zhao et al., 2015
		+AKT1	X	X	X	25% cTnT/αMHC-GFP^+^ (MEFs); 5% cTnT/αMHC-GFP^+^ (CFs); 5% cTnT/αMHC-GFP^+^ (TTFs)	50% of seeded MEFs at Day 21; 0.8% of seeded CFs; 0.5% of seeded TTFs	N/A	Zhou et al., 2015
		+AKT1 and γ-secretase inhibition	X	X		70% α-actinin+, 65% cTnT+ (MEFs)	40% of seeded MEFs at Day 18	N/A	Abad et al., 2017
		+AKT1 and ZNF281		X		28% cTnT/αMHC-GFP^+^	10% of seeded TTFs after 4 weeks	N/A	Zhou et al., 2017
		None	X			13-16% cTnT^+^	~4-fold enhanced vs. GMT	N/A	Umei et al., 2017
		Sendai viral delivery		X		22% cTnT^+^	~20-fold enhanced by sendai viral delivery vs. retroviral	N/A	Miyamoto et al., 2018
		+AKT1 and EGFR/JAK inhibition	X	X	X	32% cTnT/αMHC-GFP^+^ (MEFs); 20% cTnT/αMHC-GFP^+^ (CFs); 20% cTnT/αMHC-GFP^+^ (TTFs)	~2-fold enhanced by EGFR/JAK inhibition	N/A	Hashimoto et al., 2019
		Polycistronic delivery +TGF-β inhibition	X			80% α-actinin^+^	~8-fold enhanced vs. GMT	N/A	Zhang et al., 2019a
		Polycistronic delivery +TGF-β inhibition	X			25% Titin-eGFP/α-actinin^+^	~6-fold enhanced vs. GMT	N/A	Zhang et al., 2019b
		Alternatively spliced MEF2C isoforms	X	X	X	5% cTnT/αMHC-GFP^+^ (MEFs), 1.8% cTnT/αMHC-GFP^+^ (CFs), 1% cTnT/αMHC-GFP^+^ (TTFs)	Not discussed	N/A	Wang et al., 2020b
		+miR-1, miR-133, and SMAD7	X			80% α-actinin^+^	~40% of seeded MEFs at Day 13	N/A	Riching et al., 2021

Overexpression of Myocardin (MYOCD) alongside MEF2C and TBX5 generated similar numbers of cTnT/αMHC-GFP double positive cells as GMT in both neonatal CFs and TTFs, but also upregulated mRNA expression of *Myh6, Myl2, Actc1, Nkx2-5*, and *Scn5a* whereas GMT only upregulated expression of *Myh6* and modestly upregulated *Actc1* expression (Protze et al., 2012). Addition of NKX2.5 to GHMT greatly improved the percentage of cells exhibiting calcium transients compared to GMT alone (1.6% vs. 0.03% in MEFs, 4.5% vs. 1% in adult CFs) (Addis et al., 2013). Expression of a MyoD transactivation domain—MEF2C (MM_3_) fusion protein alongside GHT (MM_3_-GHT) increased the generation of beating cells by 18-fold compared to GHMT in MEFs (3.2–3.5% vs. 0.2% based on the number of cells seeded) and generated detectable numbers of beating cells in neonatal TTFs. Furthermore, MM_3_-GHT increased the number of cTnT positive cells compared to GHMT (20.9% vs. 12.6% in MEFs, 29.3% vs. 11.2% in TTFs) (Hirai et al., 2013). Addition of MYOCD, Serum response factor (SRF), Mesoderm Posterior BHLH Transcription Factor 1 (MESP1), and the SWItch/sucrose non fermentable (SWI/SNF) complex subunit BAF60C (encoded by *Smarcd3*) to GMT enhanced expression of the αMHC-GFP reporter as well as *Actc1, Tnnt2, Myl2, Myh6*, and *Myh7* mRNA expression in MEFs compared to GMT alone (Christoforou et al., 2013).

**Figure 2:**
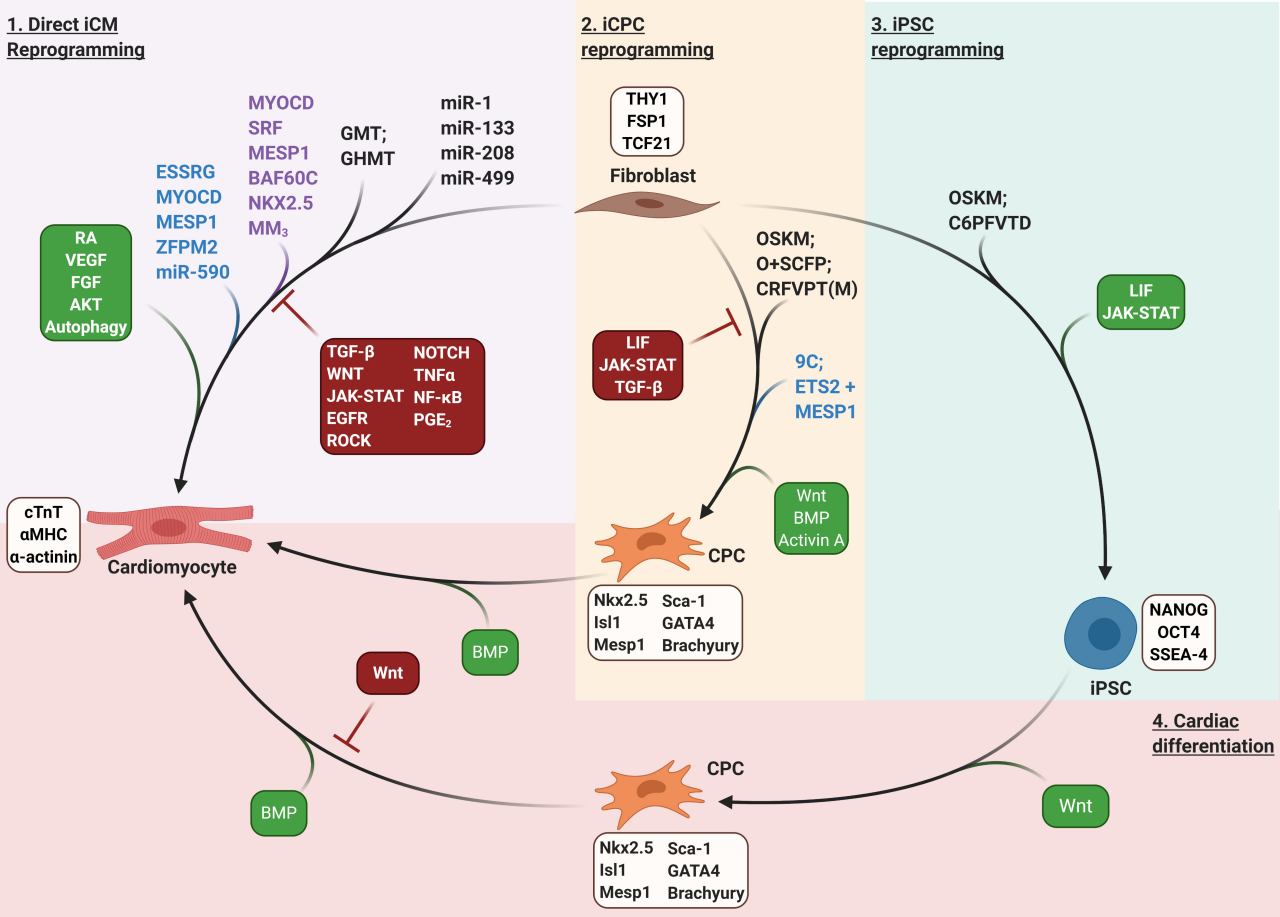
Fibroblast reprogramming strategies. Purple shaded region: direct iCM reprogramming. GMT/GHMT, *G*ATA4, *M*EF2C, *T*BX5/*G*ATA4, *H*AND2, *M*EF2C, *T*BX5; MM_3_, MYOD transactivation domain—MEF2C fusion protein. Tan shaded region: iCPC reprogramming; OSKM, *O*CT4, *S*OX2, *K*LF4, C-*M*YC; O+SCFP, *O*CT4 + *S*B431542, *C*HIR99021, *F*orskolin, and *P*arnate; CRFVPT(M), *C*HIR99021, *R*epSox, *F*orskolin, *V*alproic acid, *P*arnate, *T*TNPB, and (Rolipra*m*); 9C; CHIR99021, BIX01294, A-83-01, AS8351, SC1, OAC2, Y27632, SU16F, and JNJ10198409. Green shaded region: iPSC reprogramming. C6PFVTD; *C*HIR99021, *6*16452, *P*arnate, *F*orskolin, *V*alproic Acid, *T*TNPB, and *D*Znep. Red shaded region: cardiac differentiation of pluripotent or multipotent stem cells to generate CMs. Lineage specific markers for each cell type are listed in light brown boxes. Purple text indicates factors that enhance reprogramming of rodent fibroblasts. Blue text indicates factors that enhance reprogramming of human fibroblasts. Signaling pathways that enhance or inhibit reprogramming are depicted in green or red boxes, respectively.

Alternative reprogramming strategies have also been shown to generate induced CPCs (iCPCs), which are then capable of undergoing differentiation toward the CM lineage. Generation of multipotent iCPCs may offer additional benefits for heart regeneration as they have the potential to differentiate into endothelial and smooth muscle cells, thereby promoting vasculogenesis. Further, while iCMs rapidly exit the cell cycle upon reprogramming, iCPCs remain proliferative until they differentiate into CMs (reviewed in Klose et al., 2019; Lopez-Muneta et al., 2020). Overexpression of OCT4, KLF4, SOX2, and C-MYC in MEFs, which is well-known to generate iPSCs (Takahashi and Yamanaka, 2006; Takahashi et al., 2007), was observed to generate small clusters of contracting colonies by day 18 in the absence of leukemia inhibitor factor (LIF) (Efe et al., 2011). Notably, the emergence of contracting colonies was too early for cells to reprogram to iPSCs and then differentiate to CMs. Instead, Efe et al. observed that pluripotency markers including *Nanog* were never upregulated, but induction of cardiac progenitor genes *Mesp1, Isl1*, and *Gata4* occurred after 1 week suggesting reprogramming bypassed pluripotency and generated iCPCs. Inhibition of the JAK-STAT pathway and BMP4 stimulation starkly enhanced the generation of cardiac progenitors, resulting in ~80-fold more contracting colonies than TF overexpression alone (Efe et al., 2011). Moreover, the authors found that removal of C-MYC did not negatively influence reprogramming toward cardiac lineages. Strikingly, KLF4 and SOX2 overexpression could be replaced by the addition of small molecules *S*B431542 (TGF-β inhibitor), *C*HIR99021 (GSK3β inhibitor/WNT activator), *p*arnate (monoamine oxidase inhibitor), and *f* orskolin (adenylyl cyclase agonist) (SCPF), resulting in the generation of cardiac progenitors from overexpression of OCT4 alone alongside SCPF treatment (Wang et al., 2014). Treating MEFs with the combination of CHIR99021, 616452 (TGF-β inhibitor), parnate, forskolin, valproic acid (VPA, class I histone deacetylase inhibitor), TTNPB (synthetic RA analog), and DZNep (epigenetic modulator) resulted in the generation of iPSCs in the absence of TF overexpression (Hou et al., 2013). However, omitting DZNep and bFGF and treating MEFs with only *C*HIR99021, *R*epSox (TGF-β inhibitor), *f* orskolin, *V*PA, *p*arnate, and *T*TNPB (CRFVPT) instead resulted in the generation of beating colonies that bypassed pluripotency and expressed cardiac precursor markers including *Sca-1, Abcg2, Wt1, Flk1*, and *Mesp1* (Fu et al., 2015). Treatment of neonatal TTFs with CRFVPT also generated beating colonies, but to a lesser extent than MEFs. Inclusion of rolipram (PDE4 inhibitor) increased the yield of beating colonies in TTFs by >2-fold compared to CRFVPT alone (Fu et al., 2015). Strategies to promote iCM, iCPC, and iPSC reprogramming are illustrated in Figure 2.

#### Reprogramming via miRs

As an alternative to transcription factor overexpression, several groups explored microRNA (miR) delivery to drive cardiac reprogramming. The combination of miR-1, miR-133, miR-208, and miR-499 induced expression of the αMHC-CFP reporter in 1.5 to 7.7% of neonatal CFs (Jayawardena et al., 2012). Delivery of this miR combination induced endogenous expression of GHMT in neonatal CFs indicating miRs may be an alternative delivery source to upregulate expression of reprogramming factors (Dal-Pra et al., 2017). The addition of miRs to TF cocktails have also led to increased reprogramming efficiency. The addition of miR-133 to GMT generated 6-fold more calcium transients and 7-fold more spontaneously contracting cells than GMT alone, and accelerated the reprogramming process by 2 weeks in MEFs. Moreover, GMT + miR-133 increased the percentage of cells expressing sarcomere proteins α-actinin and cTnT by 4- to 7-fold vs. GMT in both MEFs and adult CFs (Muraoka et al., 2014). Similarly, addition of miR-1 and miR-133 to GHMT dramatically increased the percentage of spontaneously contracting cells vs. GHMT alone (~12% vs. <0.5%) (Zhao et al., 2015). This increase in reprogramming efficiency induced by miR-1 and miR-133 was accompanied by significant upregulation of cardiac gene signatures and a concomitant repression of pro-fibrotic genes. Non-GMT/GHMT based reprogramming strategies are summarized in Table 3.

**Table 3:** *In vitro* and *in vivo* efficiencies of non-GMT/GHMT-based reprogramming strategies.

**Reprogramming strategy**			**Fibroblast origin**			**Efficiency**			**Study**
**Species**	**Core factors**	**Additional factors**	**Embryonic**	**Dermal**	**Cardiac**	**Cardiac marker expression**	**Spontaneously beating cells**	**Reprogramming *in vivo***	**Authors**
Mouse	miRs	miR-1, miR-133, miR-208, miR-499 +JAK inhibition			X	13.4–28% αMHC-GFP^+^	1% of total cells	~1% cTnT/Fsp1-TdTomato double positive	Jayawardena et al., 2012
		miR-1, miR-133, miR-208, miR-499			X	N/A	N/A	miR combination vs. miR negative control	Jayawardena et al., 2015
								FS = 30% VS. 20% at 12 weeks; Fibrotic area = 10% vs. 25%	
	Non-GMT/GHMT Transcription factors	MYOCD, MEF2C, TBX5	X	X	X	2.5% cTnT/αMHC-TdTomato^+^	Rare	N/A	Protze et al., 2012
		OCT4, KLF4, SOX2, C-MYC + JAK inhibition and BMP4 stimulation	X	X		39% cTnT^+^ (MEFs)	120% of seeded cells, ~37% of total cells (MEFs)	N/A	Efe et al., 2011
	Small Molecules	CHIR99021, RepSox, Forskolin, VPA, Parnate, and TTNPB (CRFVPT); CRFVPT + rolipram (CRFVPTM); LIF after day 16	X	X		14.5% α-actinin^+^, 9% αMHC^+^ (MEFs)	~100 beating clusters from 50,000 starting cells (MEFs); Generation of beating cells ~2-fold enhanced by rolipram (TTFs)	N/A	Fu et al., 2015
		CRFVPTM			X	N/A	N/A	CRFVPTM vs. vehicle;	Huang et al., 2018
								EF = ~35% vs. ~23%; FS = ~18% vs. ~10% after 7 weeks	
		CRFVPTM + PTC-209	X		X	42% αMHC^+^ (MEFs), 10% αMHC^+^ (CFs)	~20% enhanced by PTC-209	N/A	Testa et al., 2020

#### Ensuring Expression and Optimal Stoichiometry of Reprogramming Factors

The increasing number of factors required to promote fibroblast reprogramming generated concern that simultaneous transduction of three or more viral vectors into the same cell was inefficient and could represent a technical barrier to achieving optimal reprogramming. Thus, polycistronic expression vectors containing GMT separated by “self-cleaving” 2A peptides were developed to ensure all three reprogramming factors were transduced into the same cell (Inagawa et al., 2012). Transduction of polycistronic GMT nearly doubled the percentage of adult rat CFs expressing cTnT compared to individual G/M/T delivery (7.5% vs. 4%) (Mathison et al., 2014). An inherent property of polycistronic expression systems is that the splicing order of genes affects protein expression levels. Thus, polycistronic vectors containing all possible splicing orders of GMT were developed to determine the optimal expression levels for each reprogramming factor. Combinations that resulted in relatively higher levels of MEF2C and lower levels of GATA4 and TBX5 (M-G-T, M-T-G) induced expression of cTnT and the αMHC-GFP reporter in ~3–4% of reprogrammed neonatal CFs. In contrast, individual G/M/T overexpression resulted in expression of cTnT and the αMHC-GFP reporter in only ~0.3% of cells, and splicing orders that resulted in low MEF2C expression produced even lower percentages of cTnT and αMHC-GFP positive cells than individual G/M/T overexpression (Wang et al., 2015a,b).

Given the role of HAND2 in increasing reprogramming efficiency, quad-cistronic vectors were also generated to determine optimal expression levels for HAND2 alongside GMT. Relatively higher levels of GMT and lower levels of HAND2 (M-G-T-H/M-G-H-T) significantly improved the percentage of α-actinin positive cells reprogrammed from MEFs compared to vectors with relatively higher levels of HAND2 (H-M-G-T) or compared to M-G-T alone. Further, M-G-T-H/M-G-H-T both increased the number of sarcomere positive cells by 5-fold compared to M-G-T, but M-G-T-H resulted in a 6-fold increase in the number of beating cells vs. M-G-T whereas M-G-H-T only modestly increased the number of beating cells (Zhang et al., 2019b; Zhang and Nam, 2020). Sendai viral (SeV) delivery of polycistronic G-M-T robustly increased expression of GMT reprogramming factors compared to retroviral delivery in MEFs, which correlated to a 5-fold increase in the percentage of cells expressing cTnT and nearly a 100-fold increase in the generation of contracting cells after 6 weeks in culture (Miyamoto et al., 2018). Curiously, retroviral delivery of polycistronic M-G-T did not enhance reprogramming efficiency compared to individual G/M/T (~2% cTnT positive cells vs. ~3% in G/M/T), but did moderately enhance expression of cardiac genes *Actc1* and *Myocd* in MEFs. Thus, the enhanced reprogramming efficiency by polycistronic delivery observed in previous studies may be specific to postnatal and adult cardiac and dermal fibroblasts rather than embryonic fibroblasts.

Alternative splicing of the *Mef2c* gene gives rise to expression of multiple isoforms. Recent comparative analysis identified two primary isoforms used in most previous fibroblast reprogramming studies generated by alternative splicing of exon 3. Overexpression of polycistronic M-G-T including *Mef2c* containing exon 3 α2 resulted in nearly double the percentage of cells expressing the αMHC-GFP and cTnT compared polycistronic delivery including *Mef2c* containing exon 3 α1, though this increase in reprogramming efficiency was only observed in MEFs and not CFs or TTFs (Wang et al., 2020b). This observation may help resolve the discrepancies in reprogramming efficiencies demonstrated using the same TF cocktails in different laboratories.

#### Signaling Pathways That Influence Reprogramming

**Figure 3:**
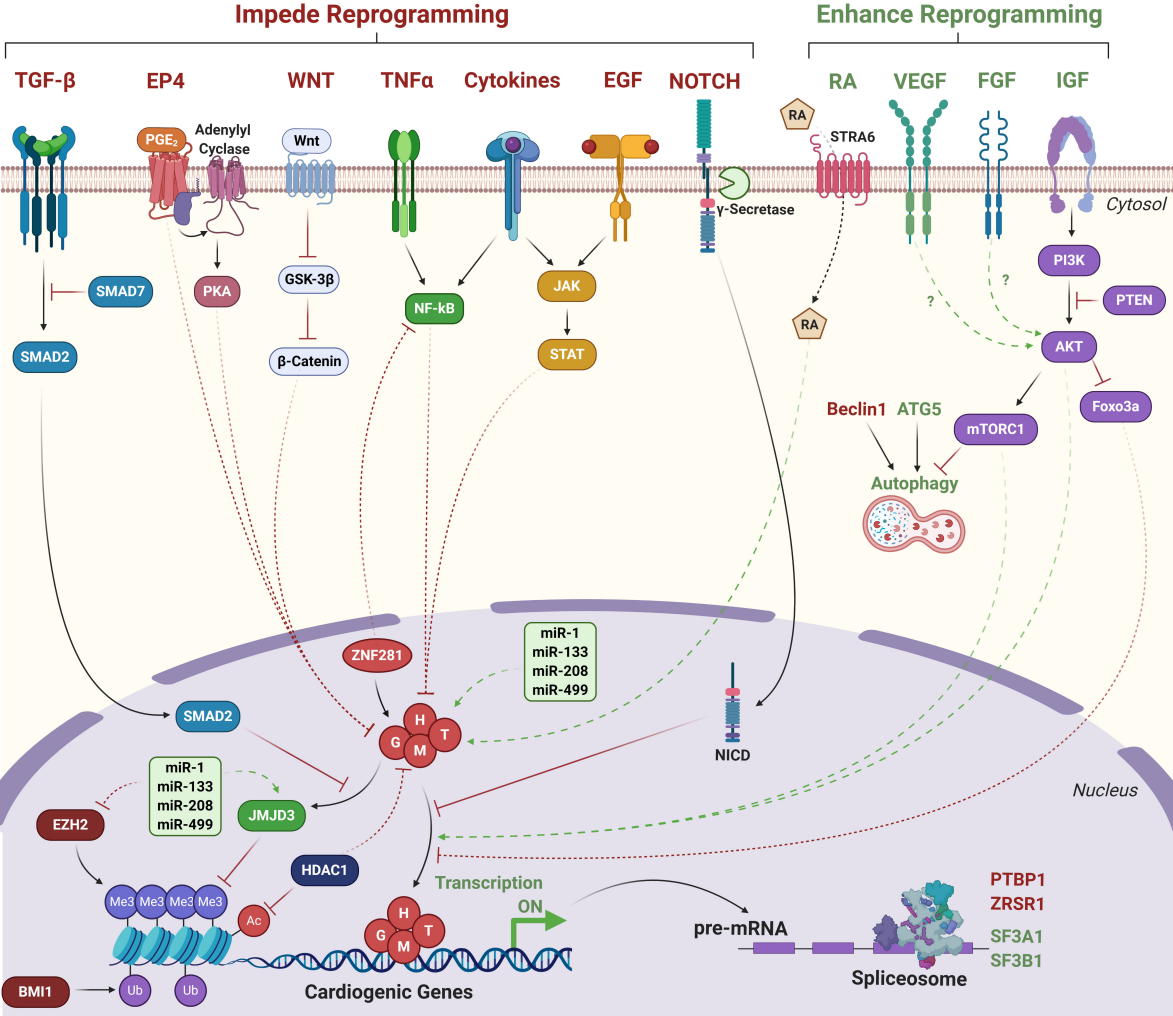
Signaling pathways that influence direct cardiac reprogramming. Green text/arrows indicate pathways that enhance reprogramming. Red text/arrows indicate pathways that negatively regulate reprogramming. Solid lines indicate direct interactions. Dashed lines indicate indirect or hypothesized interactions. G, H, M, T, GATA4, HAND2, MEF2C, TBX5; RA, Retinoic acid; NICD, Notch intracellular domain. Transcriptionally repressive histone marks: Me3; H3K27me3. Ub; H2AK119 ubiquitination. Transcriptionally activating histone marks: Ac, histone acetylation.

Numerous signaling pathways also dramatically influence the efficiency of cardiac reprogramming (Figure 3). Inhibition of the JAK-STAT pathway enhanced the percentage of reprogrammed CFs expressing αMHC-CFP by >4-fold following miR-1, miR-133, miR-208, and miR-499 delivery (Jayawardena et al., 2012). TGF-β inhibition increased the percentage of cells exhibiting calcium transients by >3-fold in both GHMT + NKX2.5- and GHMT-reprogrammed MEFs vs. DMSO control. Similarly, TGF-β inhibition or ROCK inhibition increased the generation of beating cells in MEFs transduced with GHMT + miR-1 + miR-133 by 4- to 6-fold, resulting in up to 60% beating iCMs by Day 11 (Zhao et al., 2015; Riching et al., 2018). Stimulation with TGF-β1 or TGF-β2, but not Activin A, blunted the effects of TGF-β inhibition (Ifkovits et al., 2014). Treatment with FGF2, FGF10, and VEGF (FFV) resulted in a ~20-fold increase in the number of beating cells generated by GMT transduction in MEFs compared to fetal bovine serum, and WNT inhibition alongside FFV treatment further increased the beating cell yield 4-fold vs. FFV alone (Yamakawa et al., 2015).

Dual inhibition of TGF-β and WNT signaling increased the number of contracting cells generated by polycistronic GMT overexpression in neonatal CFs by ~8-fold vs. GMT alone, resulting in ~40% contracting cells after 7 weeks of culture (Mohamed et al., 2017). Dual inhibition of histone deacetylases (HDACs) and WNT signaling alongside administration of RA increased expression of cTnT by ~7-fold compared to GMT alone in rat CFs, which coincided with increased expression of cardiac genes including *Gata4, Mef2c, Tbx5, Tnnt2, Nkx2-5*, and *Myh6* (Singh et al., 2020). Notably, though neither compound treated reprogrammed cells nor untreated cells spontaneously contracted in culture, GMT-reprogrammed cells treated with HDAC/WNT inhibitors and RA synchronously contracted following 4 week coculture with neonatal rat CMs. Knockdown of *Atg5* dramatically reduced reprogramming efficiency indicating autophagy is critical for the reprogramming process (Wang et al., 2020c). Overexpression of constitutively active AKT1 enhanced the reprogramming ability of GHMT (AGHMT) in MEFs, generating ~50% beating cells after 3 weeks of culture vs. <1% generated by GHMT alone. AKT1 also enhanced GHMT-mediated reprogramming of CFs and TTFs, though generation of beating cells was much lower (<1%) (Zhou et al., 2015).

Inhibition of γ-secretase, which cleaves NOTCH to allow nuclear translocation, in both GHMT- and AGHMT-reprogrammed fibroblasts increased the percentage of cells expressing cTnT or α-actinin (Abad et al., 2017). Notably, γ-secretase inhibition did not significantly downregulate canonical NOTCH target genes, but NOTCH is known to disrupt the ability of MEF2C to bind target genes (Wilson-Rawls et al., 1999). Indeed, γ-secretase inhibition resulted in increased MEF2C binding to *Myh6, Tnnt2*, and *Actc1* promoters (Abad et al., 2017). Addition of ZNF281 to AGHMT increased the percentage of αMHC-GFP/cTnT positive TTFs by 10-fold compared to AGHMT alone and corresponded to repressed inflammatory signaling via TNFα and NF-κB pathways (Zhou et al., 2017). Inhibition of cyclooxygenase-2 (COX-2) in GHMT- or GMT-reprogrammed postnatal or adult TTFs increased the percentage of cells expressing both αMHC-GFP and cTnT by 3- to 4-fold (Muraoka et al., 2019). Stimulation of cells with prostaglandin E2 (PGE2), activation of prostaglandin E receptor 4 (EP4), activation of PKA signaling, or administration of interleukin (IL)-1β, but not IL-6 or monocyte chemoattractant protein-1 (MCP-1), counteracted the effects of COX-2 inhibition. Notably, COX-2 inhibition did not improve reprogramming efficiency in MEFs, likely because expression of *Ptgs2* (COX-2) was much lower in MEFs than postnatal or adult TTFs, underscoring that physiological differences between fibroblast origins impact the ease of reprogramming (Muraoka et al., 2019).

Liu et al. utilized single cell RNA sequencing (scRNA-seq) following delivery of polycistronic M-G-T to identify additional barriers to cardiac reprogramming. They observed significant downregulation of genes involved in mRNA processing and splicing. Further analysis revealed that knockdown of the splicing factor *Ptbp1* led to a ~5-fold increase in the percentage of cells expressing both αMHC-GFP and cTnT vs. non-targeting short hairpin RNA (shRNA) control, and a concomitant transcriptomic shift toward the CM lineage. *Ptbp1* depletion resulted in significantly more alternative splicing events, the majority of which were exon skipping events (Liu et al., 2017). Similarly, knockdown of *Zrsr1*, a component of the U2 spliceosome complex improved reprogramming by polycistronic M-G-T in MEFs and CFs whereas knockdown of additional U2 spliceosome complex members *Sf3a1* and *Sf3b1* instead suppressed reprogramming efficiency (Zhou et al., 2018). These data identify RNA splicing as a novel regulator of cardiac reprogramming, though understanding precisely how altered splicing events contribute to the reprogramming process requires further investigation.

Mechanistic understanding of how these diverse signaling pathways influence the reprogramming process remains limited. Treatment with IGF1 mimicked AKT1 overexpression indicating insulin signaling activates AKT1 to drive reprogramming. In contrast, inhibition of PI3K, an upstream activator of AKT1, reduced reprogramming efficiency. AKT1 overexpression in reprogrammed cells was not associated with altered proliferation or apoptosis (Zhou et al., 2015). Inhibition of mTORC1 with rapamycin impaired reprogramming as did overexpression of Foxo3a. However, GSK3 inhibition did not affect reprogramming efficiency indicating that the downstream effectors of AKT1 are mTORC1 and Foxo3a but not GSK3. In contrast, Wang et al. observed enhanced reprogramming efficiency following rapamycin treatment, which they attributed to increased autophagy due to mTORC1 inhibition (Wang et al., 2020c). Thus, mTORC1 may exert dual roles on the reprogramming process; suppression of autophagy impairs the ability of the reprogramming cell to erase fibroblast identity by degrading and recycling fibrotic proteins whereas activation of protein translation may promote the ability of the reprogramming cell to acquire the CM identity via expression of cardiac proteins. Curiously, while knockdown of *Atg5* suppressed autophagy in reprogramming cells and impaired reprogramming efficiency, knockdown of *Beclin1*, a core component of the autophagy initiation complex, did not suppress baseline autophagy and potently increased reprogramming efficiency (Wang et al., 2020c). Thus, the precise regulation of autophagy during cardiac reprogramming requires further investigation.

Hashimoto et al. performed extensive chromatin immunoprecipitation followed by next generation sequencing (ChIP-seq) experiments in GMT, GHMT, and AGHMT-reprogrammed MEFs and revealed that inclusion of HAND2 and AKT1 increased the number of sites occupied by more than one reprogramming factor (50% co-occupancy in AGHMT, 46% in GHMT, and 33% in GMT) (Hashimoto et al., 2019). Moreover, cardiac enhancers enriched by Histone H3 acetylation at lysine 27 (H3K27ac), a mark of transcriptional activation, were highly enriched with MEF2 binding motifs. Inclusion of HAND2 and AKT1 to GMT promoted occupancy of these binding sites by multiple TFs suggesting cooperativity between MEF2C and other reprogramming factors to bind cardiac enhancer loci. TF enrichment at non-cardiac genes including the *Egfr* locus were also enhanced by AGHMT compared to GMT or GHMT. This enrichment corresponded to diminished H3K27ac signal and suppressed gene expression, suggesting silencing of EGFR signaling may promote cardiac reprogramming. Indeed, pharmacological or genetic inhibition of EGFR signaling increased AGHMT-mediated reprogramming efficiencies in MEFs, adult CFs, and adult TTFs (Hashimoto et al., 2019). EGFR signaling also results in the activation of pro-inflammatory signaling via activation of the JAK-STAT pathway, which is known to impair the reprogramming process (Efe et al., 2011; Jayawardena et al., 2012; Testa et al., 2020). Pharmacological inhibition of JAK2 enhanced reprogramming efficiency, similar to EGFR inhibition (Hashimoto et al., 2019). Further investigation is required to understand mechanistically how crosstalk between these diverse signaling pathways influences cardiac reprogramming. Moreover, while AKT1 facilitates enhanced TF cooperativity to bind and activate cardiac genes and repress non-cardiac genes, it is unclear what signaling downstream of mTORC1/Foxo3a mediate GHMT recruitment to target gene binding sites (Figure 3).

#### Epigenetic Repatterning in Cardiac Reprogramming

Global changes in activating and silencing epigenetic marks are well-established hallmarks of cardiomyogenesis. Marks associated with transcriptional repression including trimethylation of Lysine 27 on Histone H3 (H3K27me3) are often found within cardiac gene promoters and enhancers in pluripotent stem cells. Upon differentiation to cardiac progenitors and CMs, repressive marks are removed, and marks associated with transcriptional activation including H3K27ac and trimethylation of Histone H3 Lysine 4 (H3K4me3) are deposited at these promoters instead (Paige et al., 2012; Wamstad et al., 2012). Similarly, cardiac genes contain high levels of DNA methylation and H3K27me3 in fibroblasts, but upon reprogramming, DNA methylation and H3K27me3 are removed, followed by deposition of H3K4me3 (Ieda et al., 2010; Efe et al., 2011; Fu et al., 2013; Wang et al., 2014; Cao et al., 2016; Liu et al., 2016; Dal-Pra et al., 2017; Riching et al., 2021). In contrast, fibrotic genes contain high levels of H3K4me3 in fibroblasts. As reprogramming progresses, H3K4 is demethylated and these genes accumulate H3K27me3, albeit at a slower rate than the repatterning observed at cardiac genes (Fu et al., 2013; Cao et al., 2016; Liu et al., 2016).

Knockdown of polycomb repressive complex 1 component *Bmi1* facilitated removal of the transcriptionally repressive mark Histone H2A Lysine 119 ubiquitination (H2AK119ub) at cardiac genes and corresponded to increased H3K4me3 levels, increased expression, and increased reprogramming efficiency by polycistronic M-G-T (Zhou et al., 2016). Furthermore, 24 h pretreatment of MEFs or CFs with the Bmi1 inhibitor PTC-209 repressed STAT3-, IL-6-, and ERK1/2-mediated inflammatory signaling and resulted in a ~25% increase in the number of αMHC-GFP positive cells generated by the CRFVPT small molecule combination (Testa et al., 2020). Targeted depletion of additional epigenetic regulators including lysine-specific methyltransferases *Kmt2a, Kmt2b*, and *Kmt2e* via shRNA delivery impaired the reprogramming process whereas depletion of chromatin remodeler complex and cohesion complex subunits *Ruvbl1/Bcor* and *Stag2*, respectively, enhanced reprogramming by polycistronic M-G-T (Zhou et al., 2018). HDAC inhibition alone or in conjunction with WNT inhibition and RA stimulation improved reprogramming by GMT, which coincided with elevated global H3K27ac levels in rat CFs (Singh et al., 2020). Further, overexpression of HDAC1 counteracted the effects of HDAC inhibition, indicating that HDACs antagonize reprogramming by GMT (Figure 3).

Changes in the epigenetic landscape are controlled by balancing enzymatic activities of epigenetic writers, erasers, and readers. H3K27me3 is deposited by enhancer of zeste homolog 1 and 2 (EZH1, EZH2) and removed by ubiquitously transcribed tetratricopeptide repeat protein X-linked transcript (UTX, also known as lysine demethylase 6A, KDM6A) or Jumonji domain-containing protein D3 (JMJD3, also known as KDM6B) (Hyun et al., 2017). Pharmacological inhibition of EZH2 methyltransferase activity approximately doubled the number of spontaneously beating iCMs reprogrammed by MM_3_-GHT in MEFs (Hirai and Kikyo, 2014). Overexpression of miR-1, miR-133, miR-208, and miR-499 downregulated expression of *Ezh2* but upregulated expression of *Kdm6a* in neonatal CFs (Dal-Pra et al., 2017). Moreover, double knockdown of *Kdm6a* and *Kdm6b* prevented upregulation of *Gata4, Mef2c*, and *Tbx5* mRNA expression by miR combination, thereby impairing the reprogramming process (Dal-Pra et al., 2017). In agreement with Dal-Pra et al., our recent study indicated that *Kdm6b* knockdown also significantly impaired reprogramming of MEFs by GHMT + miR-1, miR-133, and TGF-β inhibition (Riching et al., 2021). We further demonstrated that activation of TGF-β signaling prevents removal of H3K27me3 and that the downstream TGF-β effector SMAD2 competes with GATA4 to bind and recruit JMJD3 to cardiac gene loci to facilitate removal of H3K27me3 (Figure 3). Moreover, JMJD3 enhances the interactions between GATA4 and SWI/SNF component BRG1, thereby promoting activation of cardiac gene expression independent of H3K27 demethylase activity. These data establish a novel mechanism by which inhibition of TGF-β signaling dramatically enhances epigenetic repatterning, thereby enhancing cardiac reprogramming efficiency (Riching et al., 2021). The TGF-β pathway was also recently shown to alter the occupancy of BRD4 and RNA pol II at super enhancers in CFs (Stratton et al., 2019); thus it is likely that downstream effectors of signaling pathways including the TGF-β pathway are key to driving epigenetic changes to promote or inhibit cellular differentiation. Elucidating the broad epigenetic changes in response to altered cellular signaling may therefore lead to further improvement in cardiac reprogramming.

#### *In vivo* Reprogramming

The ability to reprogram CFs *in vitro* led to determining if reprogramming CFs in the heart post-MI would restore heart function. Co-injection of retroviral particles containing GMT alongside DsRed or GFP into the infarct of immunocompromised mice resulted in activation of αMHC-GFP and α-actinin expression in DsRed or GFP positive cells (Ieda et al., 2010; Inagawa et al., 2012) and increased expression of cardiac genes in cells that took up viral particles (Chen et al., 2012). Moreover, GMT injected mice and rats showed significantly higher EF and 50–75% reduced fibrosis compared to control injections (Mathison et al., 2012, 2014; Qian et al., 2012). Notably, injection of Thymosin β4 (Qian et al., 2012) or VEGF (Mathison et al., 2012, 2014, 2017) further improved these parameters compared to GMT alone. Injection of polycistronic GMT (M-G-T) doubled the number of iCMs generated which correlated to higher EF and fractional shortening (FS) parameters and lower fibrotic area compared to individual G/M/T viral particles (Ma et al., 2015). SeV delivery of polycistronic GMT and retroviral delivery of GMT both improved EF and FS and reduced fibrosis vs. GFP control, but SeV GMT delivery also slightly improved FS over retroviral GMT (Miyamoto et al., 2018). Furthermore, labeling CFs with TdTomato driven by *Tcf21*-Cre indicated that SeV-GMT resulted in triple the number of cTnT/TdTomato positive cells compared to retroviral GMT (Miyamoto et al., 2018). Dual inhibition of TGF-β and WNT signaling further enhanced reprogramming by GMT; 12 weeks post-MI, EF in mice injected with GMT and TGF-β/WNT inhibitors, GMT alone, inhibitors alone, or DsRed was ~35, ~30, ~22, or 20%, respectively. Labeling CFs with YFP driven by *Postn*-Cre indicated GMT + TGF-β/WNT inhibition resulted in ~7 times as many YFP/cTnT double positive cells as GMT alone. Moreover, RNA-seq analysis performed on isolated YFP/cTnT double positive cells indicated that GMT overexpression alongside dual inhibition of TGF-β and WNT generated more mature iCMs that clustered closer to adult CMs by principle component analysis than those generated by GMT alone (Mohamed et al., 2017). Injection of *Beclin1* haploinsufficient mice with retroviral polycistronic M-G-T resulted in significantly improved heart function and reduced fibrosis compared to wildtype animals, though both groups demonstrated improved heart function and reduced fibrosis to mice injected with DsRed control (Wang et al., 2020c).

Addition of HAND2 to GMT likewise significantly improved heart function post-MI. Mice injected with GHMT had double the FS and 70% higher EF than mice injected with GFP 3 weeks post-MI. EF continued to improve in GHMT-injected animals by 6 and 12 weeks post-MI (EF = 57% at week 12), but slightly worsened in GFP injected mice (EF = 29% at week 12). Fibrotic area was reduced ~50% in animals injected with GHMT compared to those injected with GFP (Song et al., 2012). Notably, functional parameters in GHMT-injected animals did not reach levels comparable to those in sham or unoperated mice indicating that further optimization of *in vivo* reprogramming is required to fully regenerate the heart post-MI. Regardless, the authors comment that the level of regeneration achieved in this study seems greater than expected based on the *in vitro* reprogramming efficiency of GHMT and speculate that the native environment of the intact heart may provide additional biochemical and mechanical cues that promote reprogramming *in vivo*.

*In vivo* reprogramming was also achieved by delivery of miR-1, miR-133, miR-208, and miR-499. Following coronary artery ligation, miR delivery generated 1–12% cTnT/*Fsp1*-tdTomato double positive cells (Jayawardena et al., 2012, 2015). *Fsp1*-TdTomato specifically labeled non-myocytes in the heart, the majority of which were CFs. Notably, ~4% of cells in mice injected with negmiR control also expressed cTnT/*Fsp1*-tdTomato suggesting a small degree of promoter leakiness. Injection of miR combination blunted worsening of cardiac function post-MI. After 3 months, animals injected with miR combination had 50% higher FS than those injected with negmiR control (30% vs. 20%). Fibrotic area was also reduced 60% by miR combination (Jayawardena et al., 2015).

Finally, administration of small molecules CRFVPT + rolipram (CRFVPTM) post-MI reduced fibrotic area by >50% and significantly improved EF and FS vs. vehicle control (Huang et al., 2018). Labeling non-myocytes with tdTomato driven by *Fsp1*-Cre indicated that CRFVPTM generated 0.78% cTnT/tdTomato double positive cells and 1.02% MEF2C/tdTomato double positive cells. No double positive cells were generated by vehicle control. The authors noted that some compound dosing strategies, particularly those with short administration intervals induced significant weight loss indicating compound toxicity (Huang et al., 2018). Nevertheless, longer intervals between compound administration over longer periods of time did not cause noticeable toxicity suggesting small molecule delivery may be a promising therapeutic avenue to reprogram CFs to regenerate the heart. Restoration of heart function by various *in vivo* reprogramming strategies is shown in Tables 1–3. Collectively, these studies demonstrate that cardiac reprogramming reduces fibrosis and prevents worsening of cardiac function following MI. Additionally, some studies reported improved cardiac functional parameters following MI, though incomplete, possibly due to relatively low reprogramming efficiency. Thus, these studies offer a proof of concept for regenerative therapies post-MI.

#### Translation to Human IHD

Although numerous strategies have greatly increased the efficiency of reprogramming murine fibroblasts in recent years, the efficiency of reprogramming human fibroblasts remains low. The GMT core TF cocktail, which could induce expression of the αMHC-mCherry reporter in up to ~10% of murine fibroblasts, could not induce reporter activity in human ESC-differentiated fibroblasts. However, addition of ESSRG and MESP1 could activate expression of the αMHC-mCherry reporter and cTnT. Inclusion of MYOCD and ZFPM2 further enhanced expression in ESC-derived fibroblasts and could drive expression of these markers in human dermal fibroblasts (HDFs) and human CFs (HCFs) (Fu et al., 2013). Dual inhibition of TGF-β/WNT signaling in immortalized HCFs transduced with GMT + ESSRG, MYOCD, ZFPM2, and MESP1 (7F) resulted in spontaneous calcium transients in >50% of cells after 4 weeks in culture whereas 7F expression alone generated calcium transients in <5% of reprogrammed cells (Mohamed et al., 2017). GMT + MESP1 and MYOCD (GMTMM) induced expression of cTnT and α-actinin in ~5–6% of HCFs and ~1% of these cells exhibited calcium transients (Wada et al., 2013). Though spontaneously beating cells could not be identified in reprogrammed human CFs, co-culture with rat CMs resulted in ~5% of reprogrammed cells to synchronously contract. Addition of miR-133 to GMTMM increased the percentage of cTnT positive cells by 3- to 10-fold vs. GMTMM alone (Muraoka et al., 2014). Overexpression of GHT, MYOCD, miR-1, and miR-133 induced cTnT or tropomyosin expression in >35% of human foreskin fibroblasts (HFFs) after 4 weeks. This combination of reprogramming factors also induced expression of cTnT in HCFs and HDFs, albeit at lower efficiencies. Additionally, after 11 weeks in culture, rare beating cells were observed in reprogrammed HCFs, but not in reprogrammed HFFs or HDFs (Nam et al., 2013). GHMT + MYOCD or GMT + miR-590 both induced expression of cTnT in ~5% of HCFs (Singh et al., 2016). Dual inhibition of HDACs and WNT alongside administration of RA further improved reprogramming by GHMT, MYOCD, and miR-590 compared to vehicle control (25% vs. 5.7% cTnT positive cells) (Singh et al., 2020). Though neither reprogramming condition generated spontaneously beating cells, ~5% of reprogrammed cells treated with these compounds synchronously contracted after 4 weeks of coculture with neonatal rat CMs. SeV delivery of GMTMM + miR-133 induced expression of cTnT in ~15% of HCFs after 10 days in culture (Miyamoto et al., 2018). Using a nuclease-dead Cas9 (dCas9) fused to the VP64 transcriptional activator (dCas9-VP64) alongside delivery of sgRNAs targeting *GATA4, HAND2, MEF2C, TBX5*, and *MEIS1* loci, Wang et al. demonstrated robust activation of endogenous TF expression resulting in cTnT expression in ~9% of HFFs (Wang et al., 2020a).

Though less efficient than other methods, delivery of polycistronic M-G-T + miR-133 to ESC-derived fibroblasts generated ~2.5% cTnT positive cells (Zhou et al., 2019). Zhou et al. then utilized puromycin selection following M-G-T + miR-133 transduction in ESC-derived fibroblasts and performed scRNA-seq to identify barriers to human reprogramming. This analysis suggested that high expression level of reprogramming factors correlated to more complete reprogramming and cell cycle exit whereas lower expression of reprogramming factors resulted in reversion back to a fibroblast state. The rate at which human fibroblasts reprogram was noted to be much slower than murine fibroblasts. By day 3, GMT/miR-133 induced differential expression of only 1/3rd the number of genes in human fibroblasts compared to murine fibroblasts (Liu et al., 2017; Zhou et al., 2019). Moreover, delivery of reprogramming factors generated an innate immune response, which was crucial to the reprogramming process. Knockdown of *TLR3, NFKB1*, or *PTGS2* all lowered the percentage of cTnT positive cells, in part by increasing DNA methylation at cardiac genes (Zhou et al., 2019). Intriguingly, inhibition of COX-2 (*PTGS2)* promoted reprogramming efficiency in mouse dermal fibroblasts (Muraoka et al., 2019) suggesting that inflammatory signaling may have vastly different effects on reprogramming efficiencies in different species.

Non-GMT or GHMT-based methods have also successfully generated iCPCs from human fibroblasts. Overexpression of E26 transformation-specific transcription factor 2 (ETS2) and MESP1 followed by treatment with Activin A and BMP4 generated CPCs marked by NKX2.5 expression from HDFs, which progressed through cardiac differentiation to CM-like cells, marked by sarcomere proteins including α-actinin and α-MHC (Islas et al., 2012). Finally, treatment with CHIR99021, BIX01294 (H3K9 methyltransferase inhibitor), A-83-01 (TGF-β inhibitor), AS8351 (H3K4 demethylase inhibitor), SC1 (ERK1 inhibitor), OAC2 (OCT4 activator), Y27632 (ROCK inhibitor), SU16F (PDGFRβ inhibitor), and JNJ10198409 (PDGFRβ and PDGFRα inhibitor) (9C) could also induce differentiation of HFFs or human fetal lung fibroblasts (HLFs) to iCPCs. Cao et al. observed that ~6.6% of HFFs or HLFs treated with 9C expressed cTnT by day 30, with beating clusters of cells appearing starting at day 20 (Cao et al., 2016). Fibroblasts treated with 9C did not upregulate pluripotency markers but did express mesodermal/cardiac progenitor markers including *T* (Brachyury), *MESP1, GATA4, NKX2-5*, and *KDR*. Furthermore, H3K4me3 was lost at fibrotic genes and gained at cardiac genes whereas H3K27me3 was lost at cardiac genes and gained at fibrotic genes and 9C treatment was associated with global decondensation of chromatin (Cao et al., 2016). Thus, despite relatively lower efficiencies compared to rodent reprogramming and only rare generation of beating cells, these studies, summarized in Table 4, demonstrate that human fibroblasts are capable of being reprogrammed into iCMs.

**Table 4:** Reprogramming efficiencies in human fibroblasts.

**Reprogramming strategy**		**Fibroblast origin**			**Efficiency**		**Study**
**Core factors**	**Additional factors**	**Embryonic**	**Dermal**	**Cardiac**	**Cardiac marker expression**	**Spontaneously beating cells**	**Authors**
GMT	+ESSRG, MESP1, MyoCD, and ZFPM2	X	X	X	13% cTnT/αMHC-GFP^+^ (ESC-fibroblasts); 3.7% cTnT^+^ (HDFs); 1.8% cTnT^+^ (HCFs)	Not detected	Fu et al., 2013
	+MESP1 and MyoCD			X	5.5% α-actinin^+^, 5.9% cTnT^+^	Not detected	Wada et al., 2013
	+MESP1, MyoCD, and miR-133			X	23–27% cTnT^+^	Not discussed	Muraoka et al., 2014
	+ESSRG, MESP1, MyoCD, ZFPM2, and dual TGF-β/WNT inhibition			X	12.5% TNT-GCaMP5^+^; >50% of cells generated calcium transients	Not discussed	Mohamed et al., 2017
	Sendai viral delivery +MESP1, MyoCD, and miR-133			X	15% cTnT^+^	Not detected	Miyamoto et al., 2018
	+miR-133	X		X	2.5% cTnT^+^ (ESC-fibroblasts)	Not detected	Zhou et al., 2019
GHMT	+MyoCD or +miR-590			X	5% cTnT^+^	Not discussed	Singh et al., 2016
	+MyoCD, miR-590, HDAC/WNT inhibition, and retinoic acid			X	25% cTnT^+^	Not detected	Singh et al., 2020
	CRISPRa targeting GHMT + MEIS1		X		8.7% cTnT^+^	Not discussed	Wang et al., 2020a
GHT	+MyoCD, miR-1, and miR-133		X	X	35% cTnT^+^ (HFFs); 13% cTnT^+^ (HCFs); 9.5% cTnT^+^ (HDFs)	Rare after ~11 weeks in culture (HCFs)	Nam et al., 2013
ETS2 and MESP1	+Activin A and BMP4		X		13.7% αMHC-GFP+	Not discussed	Islas et al., 2012
Small Molecules	CHIR99021, BIX01294, A83-01, AS8351, SC1, OAC2, Y27632, SU16F, and JNJ10198409	X	X		6.6% cTnT^+^	~0.44% of total cells, 97% of αMHC-GFP^+^ cells	Cao et al., 2016

## Perspectives

Though the development of infarct revascularization techniques dramatically reduced infarct size, which coincided with substantial reduction in in-hospital mortality and improved patient outcomes post-MI, mortality rates post-MI remain high. Identifying novel targetable pathways to blunt reperfusion injury could further reduce infarct size and post-MI mortality. Additionally, significant progress has been made over the last two decades in identifying strategies to regenerate the myocardium following ischemic injury and elucidating mechanisms to enhance these strategies. Moreover, both induction of CM proliferation and direct fibroblast reprogramming have resulted in partial restoration of heart function and reduced fibrotic area in pre-clinical animal models of MI (Figure 1). However, significant challenges must be overcome before these strategies can be translated into novel therapies for human heart regeneration.

The majority of studies reviewed above utilized adenoviral, lentiviral, or retroviral delivery of genetic factors that induce CM proliferation or fibroblast reprogramming, which have been previously linked to carcinogenesis (Escors and Breckpot, 2010) and the development lethal immune reactions in human patients (Sibbald, 2001). The Giacca group utilized intramyocardial AAV-delivery of miR-199a-1 to induce CM proliferation in both rodent and porcine MI models (Eulalio et al., 2012; Gabisonia et al., 2019). In contrast to adeno-, retro-, and lentiviral infection, AAV delivery has been used in hundreds of clinical trials to date with no evidence of tumor formation in >7 years of patient follow up (Colella et al., 2018). On the other hand, delivery of AAVs that display tropism for the heart including AAV6 and AAV9 are also well-established to infect other organs including the brain, lung, liver, and skeletal muscle (Wu et al., 2006; Castle et al., 2016; Naso et al., 2017). Moreover, expression of cargo is long lasting while inducing cardiac regeneration may only require transient expression (Zacchigna et al., 2014). Thus, delivery of modified mRNAs has also been explored as a therapeutic avenue for gene therapy. Lesizza et al. demonstrated that a single injection of miR-199a-3p or miR-590-3p mimics improved survival in mice post-MI coinciding with improved ventricular function, reduced scar size, and the induction of CM proliferation (Lesizza et al., 2017).

Strong expression of pro-proliferative gene cargo such as CDK/Cyclin proteins in non-myocytes may potentially trigger expansion of these cells, leading to activation of fibrosis or uncontrolled vasculogenesis, negatively affecting heart function. Similarly, inducing uncontrolled expansion of CMs via NRG1 overexpression, *Salv* KO, constitutive YAP activation, or Cyclin A2 overexpression induced CM hyperplasia and cardiomegaly in embryonic and neonatal animals, resulting in cardiac dysfunction with potentially lethal consequences (Chaudhry et al., 2004; Heallen et al., 2011; Von Gise et al., 2012; Gemberling et al., 2015). Unlike viral delivery, the gene dosage of cargo can also be more carefully controlled via modified mRNA delivery, which may mitigate deleterious effects of strong expression, especially in non-target cells (Sultana et al., 2017).

Additional consideration should also be given to the maturation state of CMs generated by these regenerative strategies. While *in vivo* regeneration in rodent models has not been associated with the development of cardiac arrhythmia thus far, Gabisonia et al. demonstrated that the majority of pigs (7/10) that were administered AAV6-miR-199a-1 died of sudden death induced by tachyarrhythmia events/ventricular fibrillation 7 to 8 weeks after injection (Gabisonia et al., 2019). It is unclear if the cause of these arrhythmogenic phenotypes are a result of poor differentiation of newly proliferated CMs within the infarct leading to reentry currents or if the opposite strand of miR-199-3p, miR-199-5p, which has known deleterious consequences in CMs (Li et al., 2017), induces cardiac dysfunction. Understanding the long-term consequences of novel cardiac regeneration strategies, particularly in large animal pre-clinical studies is a required first step toward clinical translation. Overcoming these clinical barriers will undoubtedly begin a new era in the treatment of human heart disease.

## Data Availability Statement

The original contributions presented in the study are included in the article/supplementary material, further inquiries can be directed to the corresponding author/s.

## Author Contributions

AR and KS co-wrote the manuscript. All authors contributed to the article and approved the submitted version.

## Conflict of Interest

The authors declare that the research was conducted in the absence of any commercial or financial relationships that could be construed as a potential conflict of interest.
